# Almost Seven Decades of Coastal Bird Community Recovery Across Three European Seas

**DOI:** 10.1111/gcb.70623

**Published:** 2025-11-26

**Authors:** Carlos Cano‐Barbacil, Diana E. Bowler, Gustavo A. Ballesteros‐Pelegrín, Albert Bertolero, Klaas Deneudt, Meritxell Genovart, Miguel Ángel Gómez‐Serrano, Antonio J. Hernández‐Navarro, Daniel Oro, Antonio Zamora‐López, Peter Haase

**Affiliations:** ^1^ Senckenberg Research Institute and Natural History Museum Frankfurt Department of River Ecology and Conservation Gelnhausen Germany; ^2^ Departamento de Biodiversidad y Biología Evolutiva Museo Nacional de Ciencias Naturales, CSIC Madrid Spain; ^3^ Biodiversity Monitoring & Analysis UK Centre for Ecology & Hydrology Wallingford UK; ^4^ Faculty of Philosophy and Letters, Department of Geography Autonomous University of Madrid Madrid Spain; ^5^ ANSE‐Association of Naturalists of the Southeast Murcia Spain; ^6^ Associació Ornitològica Picampall de les Terres de l'Ebre Amposta Spain; ^7^ Flanders Marine Institute (VLIZ) InnovOcean Campus Ostend Belgium; ^8^ CEAB (CSIC), Department of Ecology and Complexity, Theoretical and Computational Ecology Group Blanes Catalonia Spain; ^9^ Cavanilles Institute of Biodiversity and Evolutionary Biology, University of Valencia Paterna Spain; ^10^ Faculty of Biology, Department of Zoology and Physical Anthropology University of Murcia Murcia Spain; ^11^ Faculty of Biology University of Duisburg‐Essen Essen Germany

**Keywords:** Baltic Sea, conservation, Mediterranean Sea, meta‐analysis, North Sea, protected areas, seabirds, time series analysis

## Abstract

Marine ecosystems have been historically impacted by human activities, leading to significant declines in biodiversity. Despite conservation efforts and the establishment of protected areas over recent decades, many marine species remain threatened. Here, we used a large‐scale database of 308 time series of coastal bird communities collected between 1957 and 2024 across three European regional seas to assess how abundance, taxonomic and functional diversity have changed over the past decades, and to evaluate the effect of conservation areas on coastal bird communities. Our results showed overall increases in taxonomic richness (1.7% per year on average), taxonomic diversity (1.4%), abundance (2.7%), functional richness (4.1%) and functional evenness (0.7%) of coastal bird communities. Although these overall increases were similar across the three seas investigated, they were not uniform within them. Recovery in the Western Mediterranean Sea occurred primarily between 1970 and 2000, while in the Baltic Sea, increases have occurred since 1995. For the Baltic Sea, we also found that taxonomic and functional richness, along with total abundance, are increasing more rapidly in wintering compared to breeding communities. Besides the overall mean increases, trends were highly variable across sites, including 4.5% of them experiencing significant declines in species richness, 5.2% in taxonomic diversity, and 13.3% in abundance. This site‐scale variability underscores the need for targeted conservation strategies that address local challenges. Our results also showed the relevance of conservation areas for coastal birds, especially those strictly protected. However, the effectiveness of protection depends on additional factors beyond the formal protection status alone. As some anthropogenic pressures persist, additional conservation actions are needed to ensure that marine bird communities continue to recover, but also to maintain stability in those populations that may have reached their carrying capacity.

## Graphical abstract

Our study examined long‐term trends in coastal bird communities across three European seas from 1957 to 2024. We found that bird abundance and diversity have increased over time, although these improvements vary among regions and sites. Protected areas—particularly strictly protected ones—play an important role in supporting these recoveries, but continued conservation efforts are needed to sustain and stabilize bird populations facing ongoing human pressures.

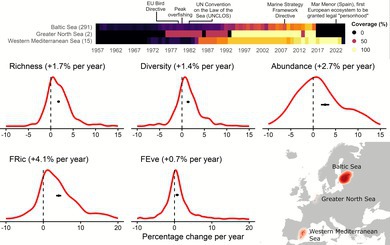

## RESUMEN

Los ecosistemas marinos han estado históricamente afectados por las actividades humanas, lo que ha provocado importantes disminuciones en la biodiversidad. A pesar de los esfuerzos de conservación y del establecimiento de áreas protegidas durante las últimas décadas, muchas especies marinas siguen estando amenazadas. En este estudio, utilizamos una base de datos a gran escala que comprende 308 series temporales de comunidades de aves costeras recopiladas entre 1957 y 2024 en tres mares europeos, con el fin de evaluar cómo han cambiado la abundancia, la diversidad taxonómica y la diversidad funcional en las últimas décadas, así como para analizar el efecto de las áreas de conservación sobre las comunidades de aves costeras. Nuestros resultados mostraron incrementos generales en la riqueza taxonómica (1.7% anual en promedio), la diversidad taxonómica (1.4%), la abundancia (2.7%), la riqueza funcional (4.1%) y la diversidad funcional (0.7%) de las comunidades de aves costeras. Aunque estos aumentos generales fueron similares en los tres mares estudiados, no fueron uniformes dentro de cada uno. La recuperación en el mar Mediterráneo occidental se produjo principalmente entre 1970 y 2000, mientras que en el mar Báltico los incrementos se observan desde 1995. En el caso del mar Báltico, también encontramos que la riqueza taxonómica y funcional, junto con la abundancia total, están aumentando más rápidamente en las comunidades invernantes que en las reproductoras. Más allá de los incrementos medios generales, las tendencias fueron muy variables entre sitios, incluyendo un 4.5% de ellos con disminuciones significativas en la riqueza de especies, un 5.2% en la diversidad taxonómica y un 13.3% en la abundancia. Esta variabilidad a escala local pone de manifiesto la necesidad de estrategias de conservación específicas que aborden los desafíos locales. Nuestros resultados también destacan la relevancia de las áreas de conservación para las aves costeras, especialmente aquellas con mayor grado de protección. Sin embargo, la efectividad de la protección depende de factores adicionales más allá del grado de protección. Dado que algunas presiones antropogénicas persisten, se requieren acciones de conservación adicionales para garantizar que las comunidades de aves marinas continúen recuperándose y, al mismo tiempo, mantener la estabilidad en aquellas poblaciones que podrían haber alcanzado su capacidad de carga.

## 1 Introduction

The ongoing biodiversity crisis, marked by a global decline in wildlife populations and high extinction rates (Dirzo et al. 2014; Johnson et al. 2017), is affecting ecosystem function and reducing essential ecosystem services (Galetti et al. 2013; Gaston and Fuller 2008; Hooper et al. 2012; Whelan et al. 2015). In marine ecosystems, direct exploitation, climate change, pollution, sea use change, invasive alien species, bycatch and even the loss of terrestrial breeding sites are the main drivers of marine biodiversity loss (Jaureguiberry et al. 2022). Despite these threats, long‐term trends in marine biodiversity remain less documented than those of terrestrial or freshwater taxa (Blowes et al. 2019; Haase et al. 2023; Rumschlag et al. 2023), highlighting the need for continued monitoring and long‐term studies in marine habitats.

Coastal birds (i.e., shorebirds and wildfowl that inhabit coastal habitats, small islands and transitional habitats such as estuaries or salt marshes) play an important role in marine and coastal environments (Stillman and Goss‐Custard 2010). For example, they influence nutrient cycling and energy flow, linking marine and terrestrial ecosystems through their foraging and nesting activities (Croll et al. 2005). However, they are a particularly threatened group because of overfishing, bycatch mortality, changes in environmental conditions, the destruction of breeding areas and the introduction of invasive mammalian predators (e.g., rats and cats) and species that destroy their nesting habitats (e.g., rabbits) (Arcos 2011; Carroll et al. 2017; Courchamp et al. 2003; Lewison et al. 2012; Spatz et al. 2017). According to the International Union for Conservation of Nature (IUCN) Red List criteria, 31% of all marine birds (110 of 359 species) are globally threatened (i.e., Critically Endangered, Endangered or Vulnerable), and another 11% (40 species) are Near Threatened (BirdLife International 2018). Populations of wide‐ranging pelagic species and migratory species are at greater risk compared to coastal bird populations with reduced ranges due to the difficulty of implementing broad‐scale actions to reduce ocean pollution, bycatch and overfishing (Paleczny et al. 2015). In contrast, species with smaller foraging ranges, including many coastal species, are less exposed to those impacts and respond more effectively to localized measures such as invasive species removal (Paleczny et al. 2015).

Over the last few decades, conservation efforts and management actions have led to positive responses in coastal bird populations (e.g., Capizzi et al. 2016; Le Corre et al. 2015) and marine ecosystems in general (Lotze et al. 2006). In Europe, the implementation of the EU Bird Directive, the development of European Bird Species Action Plans targeting threatened species and the creation of new protected areas—such as national parks, biosphere reserves, Special Protection Areas and Ramsar sites—have played a key role in addressing the ongoing biodiversity crisis in coastal and marine environments. These efforts have contributed to stabilizing and improving the population trends of coastal and waterbirds, especially those considered to be of high conservation concern (Arslan et al. 2022; Gaget et al. 2020; Roda et al. 2025; Wauchope et al. 2022). However, a full recovery of coastal bird populations is not always guaranteed, particularly for species extirpated for extended periods, or in areas where human pressure continues to increase (Buxton et al. 2014; Doyle et al. 2020; Kawakami and Horikoshi 2022).

Coastal birds are particularly well‐studied indicators of environmental change (Burger and Gochfeld 2004; Dias et al. 2019; Piatt et al. 2007). Similar to other bird species, our capacity to monitor them across extensive spatial and temporal scales surpasses that for any other marine animals, making them an ideal study group to investigate long‐term changes (Cano‐Barbacil and Cano 2021; Rosenberg et al. 2019). Although numerous studies have assessed population trends of individual coastal bird species over extended time periods (e.g., Doyle et al. 2020; Happe et al. 2025), research addressing long‐term changes at the community level across broad spatial scales remains limited. This is largely due to the lack of robust and consistent time series data for most organism groups (but see de Felipe et al. 2024; Haase et al. 2023; Rosenberg et al. 2019). While some community‐level studies do exist, they have typically been conducted at local scales (Oro et al. 2009), highlighting the need for more comprehensive approaches (Rodríguez‐Caro et al. 2024). Thus, understanding changes in taxonomic and functional diversity is crucial, as it can reveal shifts in ecosystem functioning that species‐level trends alone may not detect, and can also help determine whether observed changes represent a general recovery at the community level or are restricted to certain species (Guillerme et al. 2025; Mouillot et al. 2013).

Here, we used a database of 308 coastal bird time series collected between 1957 and 2024 across three European regional seas (i.e., Baltic Sea, Greater North Sea and Western Mediterranean Sea) to investigate (1) how abundance, taxonomic and functional diversity have changed in recent decades; (2) the effect of both marine and terrestrial conservation areas on coastal bird community trends; and (3) potential differences in biodiversity trends between wintering and breeding bird communities. We hypothesized that coastal bird abundance, as well as taxonomic diversity, has significantly increased over the study period, based on the documented recovery of several coastal bird populations likely driven by targeted conservation efforts. In particular, the establishment of protected areas, coupled with reductions in key anthropogenic pressures and the implementation of international conservation agreements, may have supported the recovery of both widespread and threatened species. As a result, we anticipate not only higher species richness and abundance, but also a broader range of functional traits represented within these communities, reflecting an overall increase in functional diversity.

## 2 Methods

### 2.1 Coastal Bird Community Data

We compiled time series of European coastal bird communities from the European Marine Observation and Data Network (EMODnet) and a previous study analyzing multidecadal biodiversity trends in European marine, freshwater and terrestrial ecosystems (Pilotto et al. 2020). This dataset was complemented with additional time series obtained through an open data call launched in October 2023. All time series met the following criteria: (1) had a minimum number of eight sampling years (not necessarily consecutive) with one sampling event per year; (2) included abundance estimates; and (3) had consistent sampling site, season, protocol, effort and taxonomic resolution over the observation period. Sampling consisted of visual censuses of bird species present in coastal habitats (i.e., areas located between the ecotone that separates terrestrial and marine ecosystems and the edge of the continental shelf). Accordingly, we considered both species that establish breeding colonies along the coast or on small islands, and nonbreeding species that exploit coastal habitats for foraging or migration (see Table S1 for a complete list of species). Observers recorded either individuals or breeding pairs, conducting surveys along linear transects on foot or by boat. Observations were made using a telescope, binoculars or a camera, depending on visibility and distance (see Table S2 for further details on the different datasets considered and on the sampling protocols).

**FIGURE 1:**
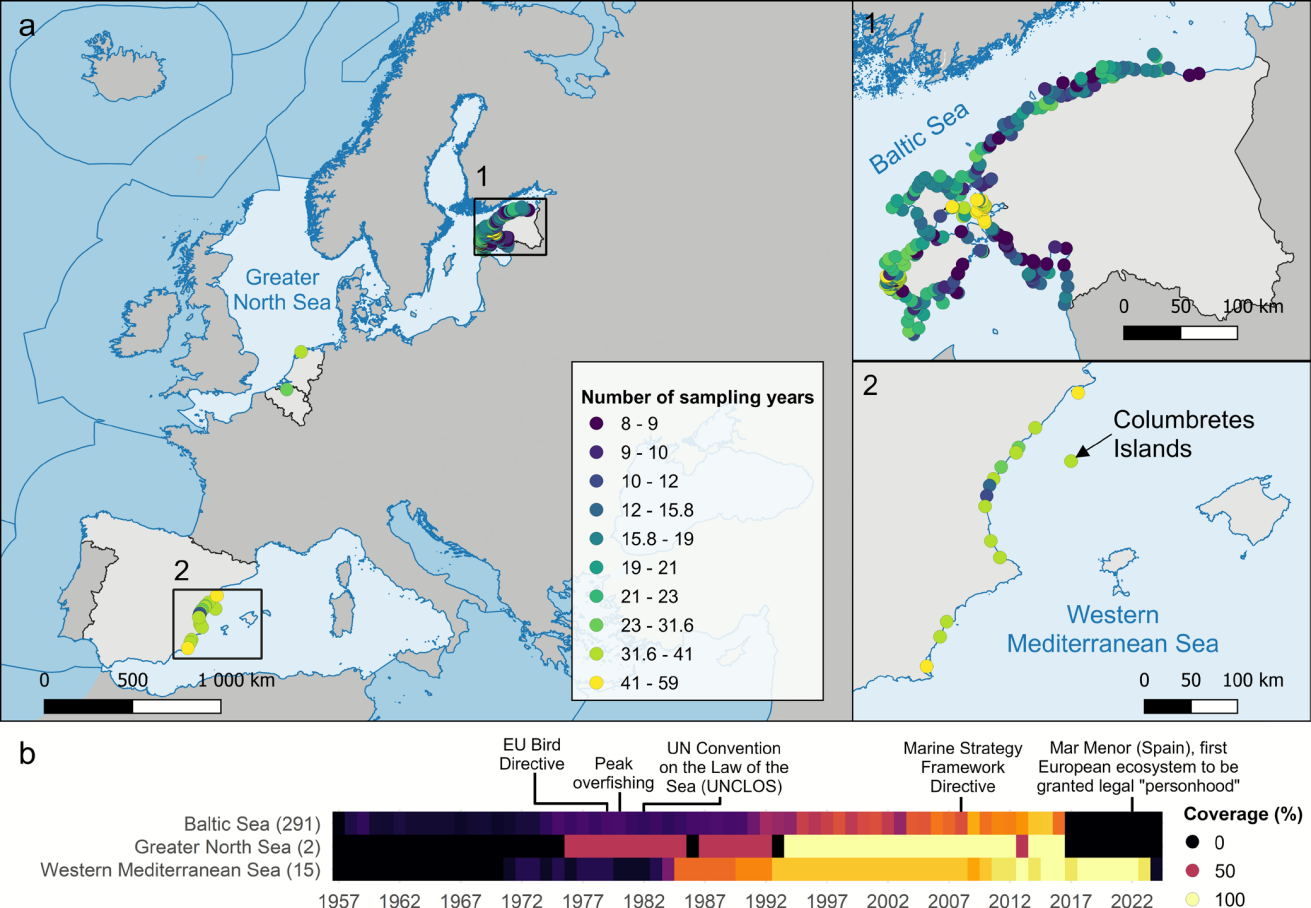
(a) Sampling sites (points) and number of sampling years (color of points) across three European regional seas (light blue) and four countries (light gray). (b) Temporal coverage across the three regional seas, with the total number of time series shown in brackets, and a timeline of major events affecting European marine ecosystems. Lighter colors indicate greater coverage. Sources: The regional seas layer for Europe was obtained from the European Environment Agency (EEA) website (version 2, October 2022), and the countries layer was obtained from Eurostat.

The final dataset comprised 56,308 observations of 121 bird taxa in 308 coastal sites (Figure 1a). Among these sites, 291 are located in the Baltic Sea (Estonia) (Estonian Ornithological Society 2019a, 2019b), two in the Greater North Sea (one in Belgium and one in the Netherlands) (Pilotto et al. 2020), and 15 in the Western Mediterranean Sea (Spain). The time series span from 1957 to 2024 with a mean total duration of 31.1 years and a mean of 21.3 sampling years (minimum 8, maximum 59 sampling years; Figure S1). However, 78% of the sampling campaigns and 75% of the observations took place between 1990 and 2024 (see Figure 1b and S2, respectively). Of the studied communities, 54.2% were sampled during the breeding season (153 in the Baltic Sea and 14 in the Western Mediterranean Sea), while the remaining 45.8% (138 in the Baltic Sea, 2 in the North Sea and 1 in the Western Mediterranean Sea) were studied during the wintering season (Table S1). All individuals were identified to the species level except in a few cases where it was not possible due to limited visibility, observational constraints, or when individuals were juveniles and difficult to distinguish. Thus, 1.3% of the total observations were identified to genus level (i.e., *Anas*, *Aythya*, *Cygnus*, *Gavia*, *Larus*, *Sterna*) and < 0.001% to family level (i.e., Anatidae). Taxa names and taxa identification numbers were harmonized using the World Register of Marine Species (WoRMS).

### 2.2 Trait Data

Trait data on European coastal birds were collected from two databases that contain comprehensive biological and ecological trait information for birds (Storchová and Hořák 2018; Tobias et al. 2022). In total we compiled data of 13 morphological and life‐history traits (see Table S3 for the complete list of traits and their definitions). We included only continuous traits for which information was available for all species. To estimate trait values for taxa that could not be identified to the species level, we calculated the mean trait values of all species within the corresponding genus or family listed in our species list.

### 2.3 Community Metrics

We calculated taxonomic richness, Shannon's diversity by using the R package “vegan” (Oksanen et al. 2024), and total abundance for each site and year. Similarly, but based on trait data, we analyzed functional diversity separately for each site and year by calculating functional richness and functional evenness using the “dbFD” function in the R package “FD” (Laliberté et al. 2014). These two metrics were chosen to complement our taxonomic approach: functional richness captures the breadth of ecological strategies present, while functional evenness conceptually parallels Shannon's index by reflecting the relative distribution of traits rather than species abundances (Mason et al. 2005). Trait data were scaled prior to the analysis to units of standard deviation.

### 2.4 Protected Areas

We collected data on the European marine and coastal (i.e., including land breeding and wintering sites) protected areas from the Protected Planet database (UNEP‐WCMC and IUCN 2024) (see Figure S3). For each site, we assessed whether it is located within a strictly protected area (SP; i.e., IUCN Categories of Protected Areas Ia, Ib or II), a less protected area (LP; i.e., IUCN Categories of Protected Areas III, IV, V, VI, or other kinds of protection) or a nonprotected area (NP; see Table S4 for detailed definitions of the different categories of protection). We also documented the year in which the site was designated as protected for all protected areas. All protected sites analyzed—both strictly and less protected—are also designated as Special Protection Areas under the Birds Directive.

### 2.5 Statistical Analysis

We used generalized least squares (GLS) models to evaluate site‐level temporal trends in abundance, and the different taxonomic and functional community metrics (see e.g., Cano‐Barbacil et al. 2025). For each site and metric, we fitted a GLS model with year as a continuous fixed effect, while accounting for temporal autocorrelation, using the R package “nlme” (Pinheiro and Bates 2023). Total abundance, taxonomic richness, and functional richness were log_10_‐transformed prior to analysis to reduce skewness. The percentage of change per year was calculated by back‐transforming model estimates. Thus, calculations varied depending on the original transformation of the response variable (see Table S5 for further details; Haase et al. 2023). To test for differences in the percentage of change in community metrics among regional seas, we used univariate permutational analysis of variance (PERMANOVA), which has the advantage of not assuming a specific probability distribution (Anderson 2017; Cano‐Barbacil et al. 2022, 2023). We used 999 permutations and Euclidean distances for the PERMANOVA. We also tested for homogeneity of dispersions for the different metrics to evaluate how uniformly these variables are distributed among the three regional seas. This analysis was performed using the function “betadisper” of the R package “vegan” (Oksanen et al. 2024).

We used generalized additive mixed models (GAMM) of the Gaussian family to evaluate nonlinear changes in abundance, taxonomic and functional community metrics over time (see e.g., Sinclair et al. 2024). The model was implemented using the “gamm” function of the “mgcv” package (Wood 2023), and included year as a smoothed fixed effect that varied by regional sea. We also included site and data source as random effects to account for site‐level variation in abundance and richness and differences in sampling protocol among data sources (Table S2). The basis dimension (k) was set to 10, which we confirmed by using the “gam.check” function of the “mgcv” package. We accounted for temporal autocorrelation by including first‐order autocorrelation between successive years sampled from the same site. Total abundance, taxonomic richness and functional richness were log_10_‐transformed prior to analysis. Additionally, we analyzed species‐level trends by using Poisson generalized linear models (GLM) and GAMMs to assess whether certain species are driving changes in the community, or conversely, if all species are responding similarly.

To evaluate the effect of protected areas on coastal bird communities, we focused our analysis on regional seas that included both protected and unprotected sites. Since all sites in the North Sea were within protected areas, this region was excluded from the analysis. We also restricted the dataset to the period between the year when the majority of currently protected sites gained protection (excluding sites that became protected later) and the final year of most time series (excluding those that ended earlier). Most protected sites were not sampled, or sampled only briefly, prior to becoming protected. We ensured sufficient data availability, requiring at least eight sampling years during the study period to calculate robust biodiversity trends. This approach enabled a standardized comparison of coastal bird community trends in strictly protected, less protected and unprotected sites over consistent time periods, without changes in protection status during the analyzed period. Consequently, our analysis included 186 sites in the Baltic Sea (*n*_SP_ = 44; *n*_LP_ = 102; *n*_NP_ = 40) and 11 sites in the Western Mediterranean Sea (*n*_SP_ = 5; *n*_LP_ = 4; *n*_NP_ = 2), spanning the periods 2005 to 2015 and 1996 to 2022, respectively. Using this reduced dataset, we calculated trends for the different metrics by using GLS models, fit separately to each site, while accounting for temporal autocorrelation. The same procedure was applied to transform the response variables and calculate the percentage of change. To assess differences in trends among strictly protected, less protected and nonprotected sites across the two seas, we used a PERMANOVA model. Protection status, sea name and their interaction were included as explanatory variables. To analyse pairwise differences among the various protection statuses, we used the “pairwise.adonis2” function of the R‐package “pairwiseAdonis” (Martinez Arbizu 2020).

Finally, to evaluate differences in biodiversity trends between wintering and breeding bird communities, we used a PERMANOVA model and tested for homogeneity of dispersions. We restricted this analysis to the Baltic Sea, as it was the only region with sufficient data for both seasons. To assess the robustness of our results, we conducted several alternative analyses. The methodology used and the results of these analyses, which were consistent with those presented in the main text, are summarized in Appendix S1. All statistical analyses were performed in R version 4.4.0 (R Core Team 2023).

## 3 Results

### 3.1 Overall Recovery of Bird Communities in Coastal Ecosystems

Across all sites, bird taxonomic richness increased by 1.7% (1.4%–2.0%; 95% confidence interval) per year, Shannon's diversity by 1.4% (1.0%–1.7%) and abundance by 2.7% (1.7%–3.6%) per year between 1957 and 2024 (Figure S4a–c). Similarly, bird functional richness increased by 4.1% (3.4%–4.8%) per year and functional evenness by 0.7% (0.3%–1.1%) per year during the study period (Figure S4d,e). Despite these overall net‐positive trends, bird taxonomic richness declined significantly at 4.5% of the studied sites, Shannon's diversity at 5.2% of sites, abundance at 13.3% of sites, functional richness at 0.6% of sites and functional evenness at 5.8% of sites.

**FIGURE 2:**
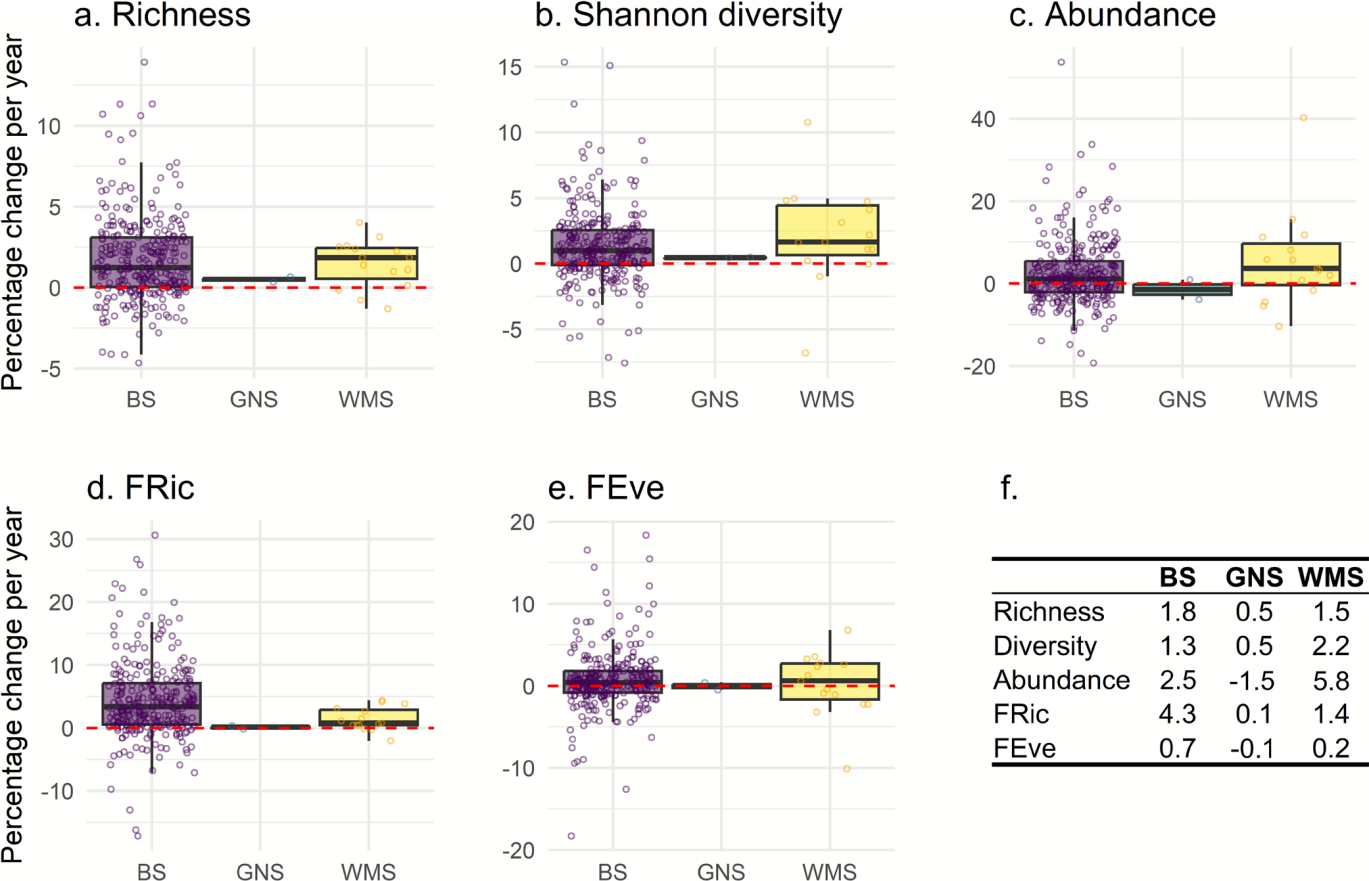
Site‐level trends by regional sea for (a) taxonomic richness, (b) Shannon diversity, (c) abundance, (d) functional richness (FRic) and (e) functional evenness (FEve) across 308 sites. Circles show the site values. Boxes correspond to the 25th and 75th percentiles; lines inside a box show the median; whiskers extend to the last observation within 1.5 times the interquartile range from the quartiles. The horizontal dashed red line shows the location of no change. (f) Summary table with the average percentages of change per year for each metric by regional sea. BS, Baltic Sea (*n* = 291); GNS, Greater North Sea (*n* = 2); WMS, Western Mediterranean Sea (*n* = 15).

Although average percentages of change showed certain variation among regional seas (Figure 2f), we did not find statistically significant differences in taxonomic richness (*R* ^2^ = 0.002; *p* = 0.701), Shannon's diversity (*R* ^2^ = 0.005; *p* = 0.442) or abundance (*R* ^2^ = 0.009; *p* = 0.242) trends among the three regional seas (Figure 2a–c). Similarly, the dispersion of richness (*F* = 2.28, df = 2, *p* = 0.081), Shannon's diversity (*F* = 1.43, df = 2, *p* = 0.205) and abundance (*F* = 1.10, df = 2, *p* = 0.243) trend values was homogeneous across regional seas. This indicates that the three studied regional seas exhibited similar variability among sites in the trends of taxonomic community metrics, and that the observed declines at certain sites were not concentrated in a single region, but rather occurred across all three seas.

In agreement with the abundance and taxonomic diversity results, we did not find significant differences in functional richness (*R* ^2^ = 0.013; *p* = 0.125) or functional evenness (*R* ^2^ = 0.001; *p* = 0.777) trends among our three regional seas (Figure 2d,e); and the dispersion of functional evenness trends was also homogeneous (*F* = 0.659, df = 2, *p* = 0.437). However, the dispersion of functional richness trends was significantly nonhomogeneous (*F* = 4.02, df = 2, *p* = 0.037), indicating greater variability among sites in the changes observed at the Baltic sites.

### 3.2 Community Recovery Was Not Homogeneous Over Time

**FIGURE 3:**
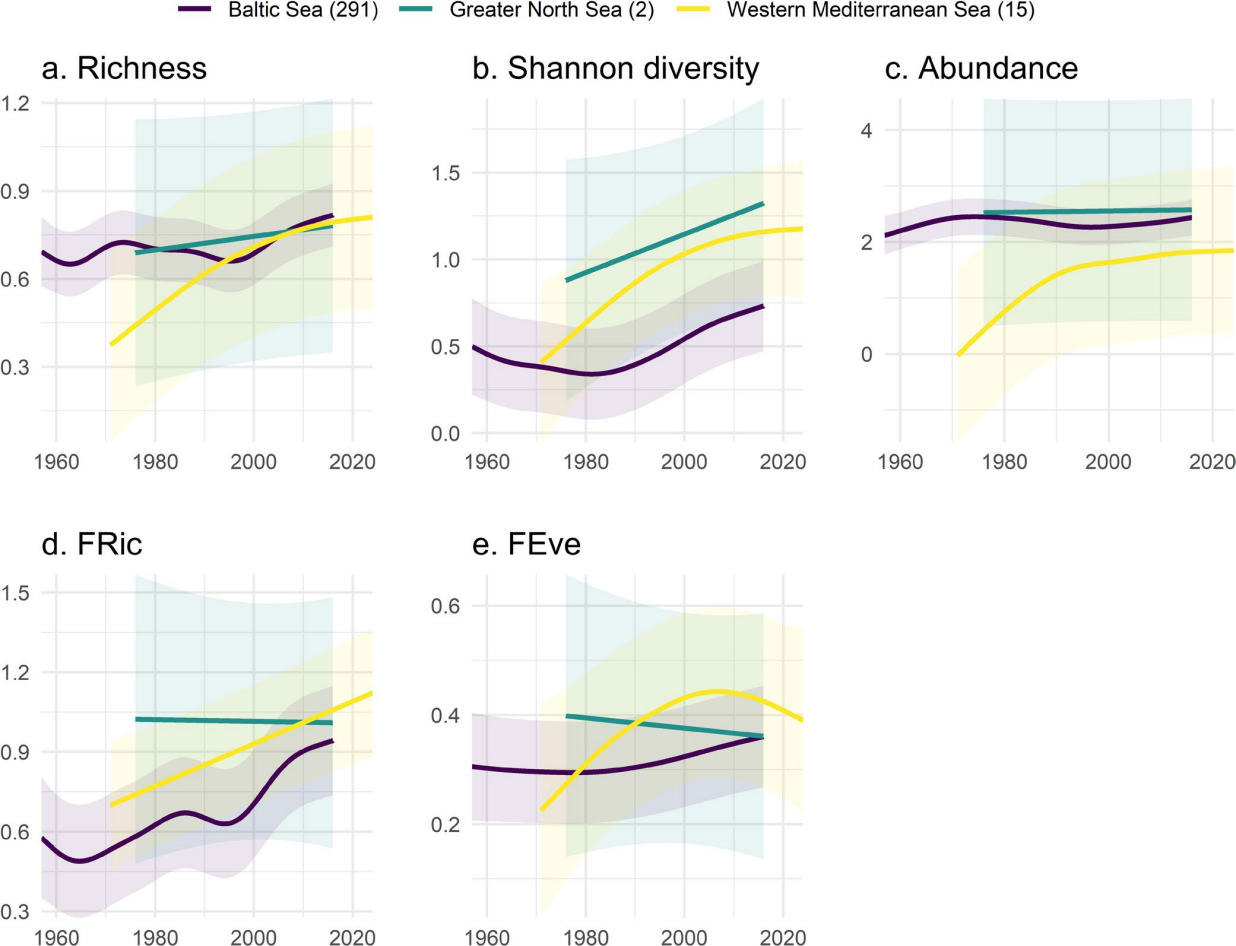
Temporal changes in (a) taxonomic richness (*R* ^2^_adj_ = 0.588), (b) Shannon diversity (*R* ^2^_adj_ = 0.437), (c) total abundance (*R* ^2^_adj_ = 0.597), (d) functional richness (FRic; *R* ^2^_adj_ = 0.494) and (e) functional evenness (FEve; *R* ^2^_adj_ = 0.243) across regional seas. The total number of time series for each regional sea is indicated in brackets. The shaded regions represent the 95% confidence intervals.

Over the past seven decades, we have observed recovery in coastal bird communities, indicated by increasing trends in local abundance, taxonomic and functional diversity. However, recovery has not occurred uniformly but has varied across regional seas including periods of greater increases interspersed with phases of stagnation or even decline (Figure 3). In the Baltic Sea, the most pronounced increases in taxonomic richness (edf = 7.0, *F* = 35.4, *p* < 0.001) and Shannon diversity (edf = 4.6, *F* = 44.0, *p* < 0.001) occurred between 1990 and 2015. In the Western Mediterranean Sea, the most significant gains in taxonomic richness (edf = 2.4, *F* = 22.2, *p* < 0.001) and Shannon diversity (edf = 2.2, *F* = 12.1, *p* < 0.001) occurred between 1970 and 2000, after which both metrics stagnated. In contrast, the Greater North Sea showed no significant changes in both metrics throughout the study period (Figure 3a,b). Total abundance in the Baltic Sea increased mainly during the early 1960s and between 2000 and 2015 (edf = 5.2, *F* = 9.4, *p* < 0.001), whereas in the Western Mediterranean Sea, the most significant gains occurred between 1970 and 1995, after which it stagnated (edf = 3.7, *F* = 18.6, *p* < 0.001). Again, no significant changes in total abundance were observed in the Greater North Sea during the study period (Figure 3c).

Similar to taxonomic metrics, functional richness (edf = 6.5, *F* = 56.8, *p* < 0.001) and evenness (edf = 2.7, *F* = 14.5, *p* < 0.001) showed the most pronounced increases in the Baltic Sea between 1990 and 2015. In the Western Mediterranean Sea, functional richness showed a steady increase (edf = 1.0, *F* = 17.7, *p* < 0.001), while the largest increases in functional evenness occurred from 1970 to 2000, followed by stagnation (edf = 2.6, *F* = 4.9, *p* = 0.014). In contrast, functional richness and evenness in the Greater North Sea showed no changes over the study period (Figure 3d,e).

Abundance changes observed at the community level were not driven by a single or a few dominant species. Instead, several species exhibited consistent recoveries over the study period, despite high variability among sites (see Figures S5–S10). For instance, in the Baltic Sea, the white‐tailed eagle ( *Haliaeetus albicilla* ; est. = 0.313, *p* = 0.027), the gadwall ( *Anas strepera* ; est. = 0.123, *p* < 0.001) and the smew ( *Mergellus albellus* ; est. = 0.113, *p* < 0.001) showed particularly marked increases in abundance. However, some of the more common species, such as the mallard ( *Anas platyrhynchos* ; est. = 0.026, *p* < 0.001) and the long‐tailed duck ( *Clangula hyemalis* ; est. = 0.036, *p* < 0.001), experienced more modest recoveries or even slight declines, as observed for the common eider ( *Somateria mollissima* ; est. = −0.050, *p* < 0.001). Similarly, in the Western Mediterranean Sea, species such as the Eurasian spoonbill ( *Platalea leucorodia* ; est. = 0.499, *p* < 0.001) or the great egret ( *Ardea alba* ; est. = 0.248, *p* < 0.001) exhibited marked increases at the study sites. Other common and widespread species, including various *Larus* species and the great cormorant ( *Phalacrocorax carbo* ; est. = 0.108, *p* < 0.001), also exhibited increasing trends over the study period. At most sites, these changes occurred primarily between the 1970s and early 2000s, consistent with the patterns observed in community‐level trends. Notably, the species showing the greatest population increase in the Greater North Sea sites was the nonnative Canada goose ( *Branta canadensis* ; est. = 0.173, *p* < 0.001).

### 3.3 Varying Trends Across Sites With Different Levels of Protection

**FIGURE 4:**
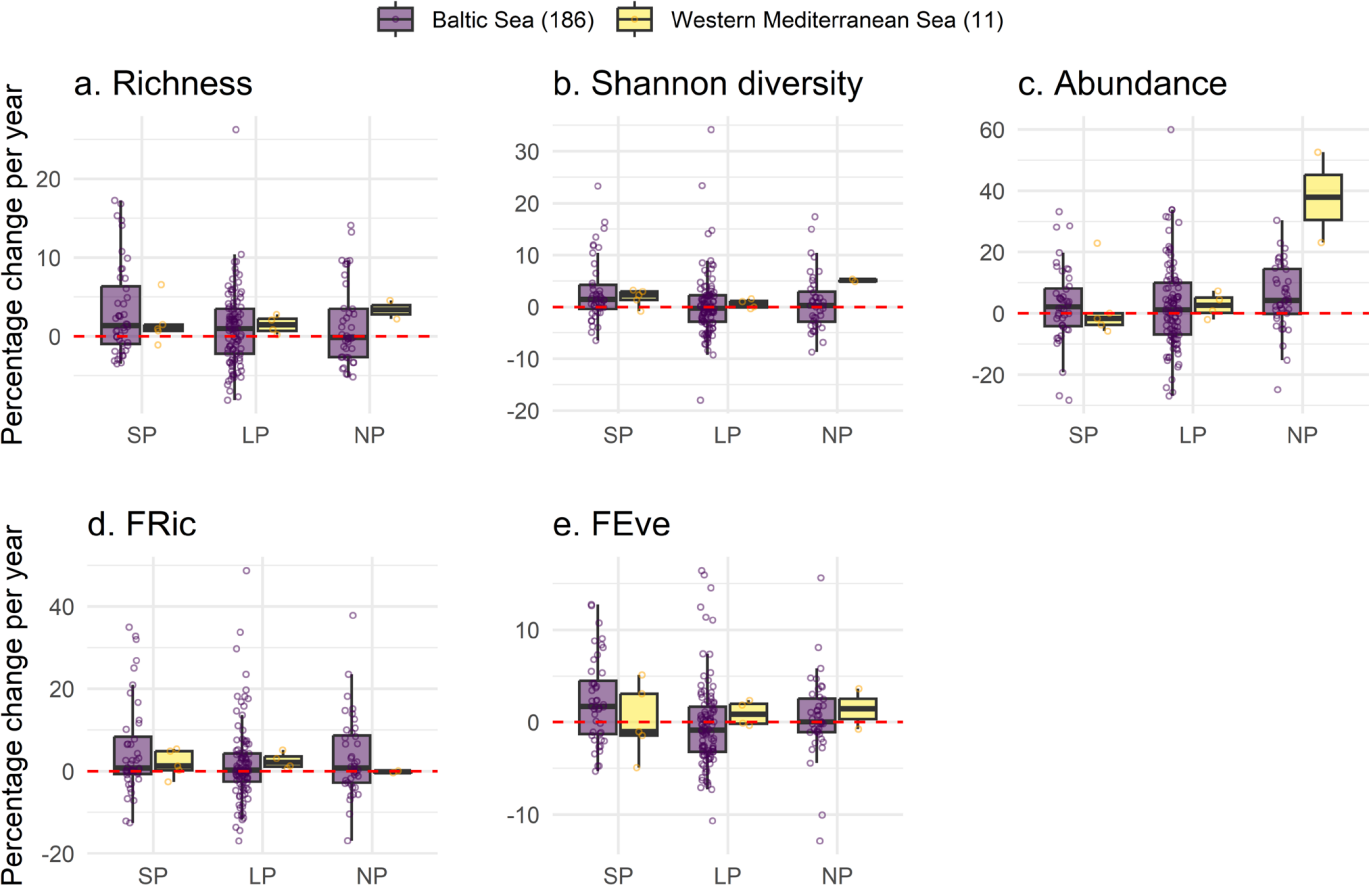
Site‐level trends by protection status and by regional sea for (a) taxonomic richness, (b) Shannon diversity, (c) abundance, (d) functional richness (FRic) and (e) functional evenness (FEve) across 197 sites. The total number of time series for each regional sea is indicated in brackets. Circles show site values. Boxes correspond to the 25th and 75th percentiles; lines inside a box show the median; whiskers extend to the last observation within 1.5 times the interquartile range from the quartiles. The horizontal dashed red line shows the location of no change. SP = Strictly protected site, IUCN Categories of Protected Areas Ia, Ib or II (*n*_BS_ = 44, *n*_WMS_ = 5); LP = Less protected site, IUCN Categories of Protected Areas III, IV, V, VI, or other kind of protection (*n*_BS_ = 102, *n*_WMS_ = 4); NP = Nonprotected site (*n*_BS_ = 40, *n*_WMS_ = 2).

The effectiveness of different levels of protection on coastal bird communities varied, with strictly protected sites (i.e., IUCN Categories of Protected Areas Ia, Ib or II) showing greater improvements in taxonomic and functional diversity metrics than less protected areas (i.e., IUCN Categories of Protected Areas III, IV, V, VI, or other kinds of protection) (Table 1 and Figure 4). More specifically, strictly protected sites showed greater increments of taxonomic richness (a mean of 3.1% versus 1.0%, pairwise *p* = 0.013), Shannon diversity (3.0% versus 0.3%, pairwise *p* = 0.011) and functional evenness (2.1% versus −0.2%, pairwise *p* = 0.004) than less protected sites. However, we did not observe differences between strictly protected and nonprotected sites and between less protected versus nonprotected sites (pairwise *p* > 0.05). Our results also revealed a more pronounced increase in the total abundance of bird communities in nonprotected areas of the Western Mediterranean Sea, supported by a significant interaction between regional seas and protection status (*p* = 0.009). This increase was greater than that observed in more protected sites in the Western Mediterranean Sea and in bird communities in the Baltic Sea.

**TABLE 1:** Results of the univariate permutational analysis of variance (PERMANOVA). Model *R* ^2^, mean percentage of change and 95% confidence interval (in brackets) for each group, degrees of freedom (df), *F* and *p* values are shown.

Response variable	Strictly protected	Less protected	Nonprotected	Explanatory variable	df	*F*	*p*
Richness *R* ^2^ = 0.032	3.1 (1.5–4.7)	1.0 (0.1–1.9)	1.6 (0.0–3.2)	Status	2	2.77	**0.049**
				Sea	1	0.01	0.954
				Status × Sea	2	0.33	0.700
Shannon diversity *R* ^2^ = 0.039	3.0 (1.3–4.7)	0.3 (−0.8–1.5)	1.1 (−0.6–2.8)	Status	2	3.30	**0.047**
				Sea	1	0.03	0.876
				Status × Sea	2	0.57	0.498
Abundance *R* ^2^ = 0.077	2.7 (−0.9–6.2)	2.4 (−0.3–5.1)	7.1 (3.0–11.2)	Status	2	2.09	0.095
				Sea	1	2.16	0.142
				Status × Sea	2	4.79	**0.009**
FRic *R* ^2^ = 0.018	4.9 (1.7–8.0)	2.0 (0.1–3.8)	3.2 (1.8–6.2)	Status	2	1.41	0.264
				Sea	1	0.38	0.548
				Status × Sea	2	0.19	0.802
FEve *R* ^2^ = 0.046	2.1 (0.8–3.4)	−0.2 (−1.1–0.7)	0.6 (−0.8–1.9)	Status	2	3.96	**0.021**
				Sea	1	0.05	0.808
				Status × Sea	2	0.61	0.476

*Note:* Statistically significant variables are highlighted in bold. Strictly protected site = IUCN Categories of Protected Areas Ia, Ib or II; Less protected site = IUCN Categories of Protected Areas III, IV, V, VI, or other kinds of protection.

Abbreviations: FEve, Functional evenness; FRic, Functional richness.

### 3.4 Contrasting Trends Between Wintering and Breeding Communities

**FIGURE 5:**
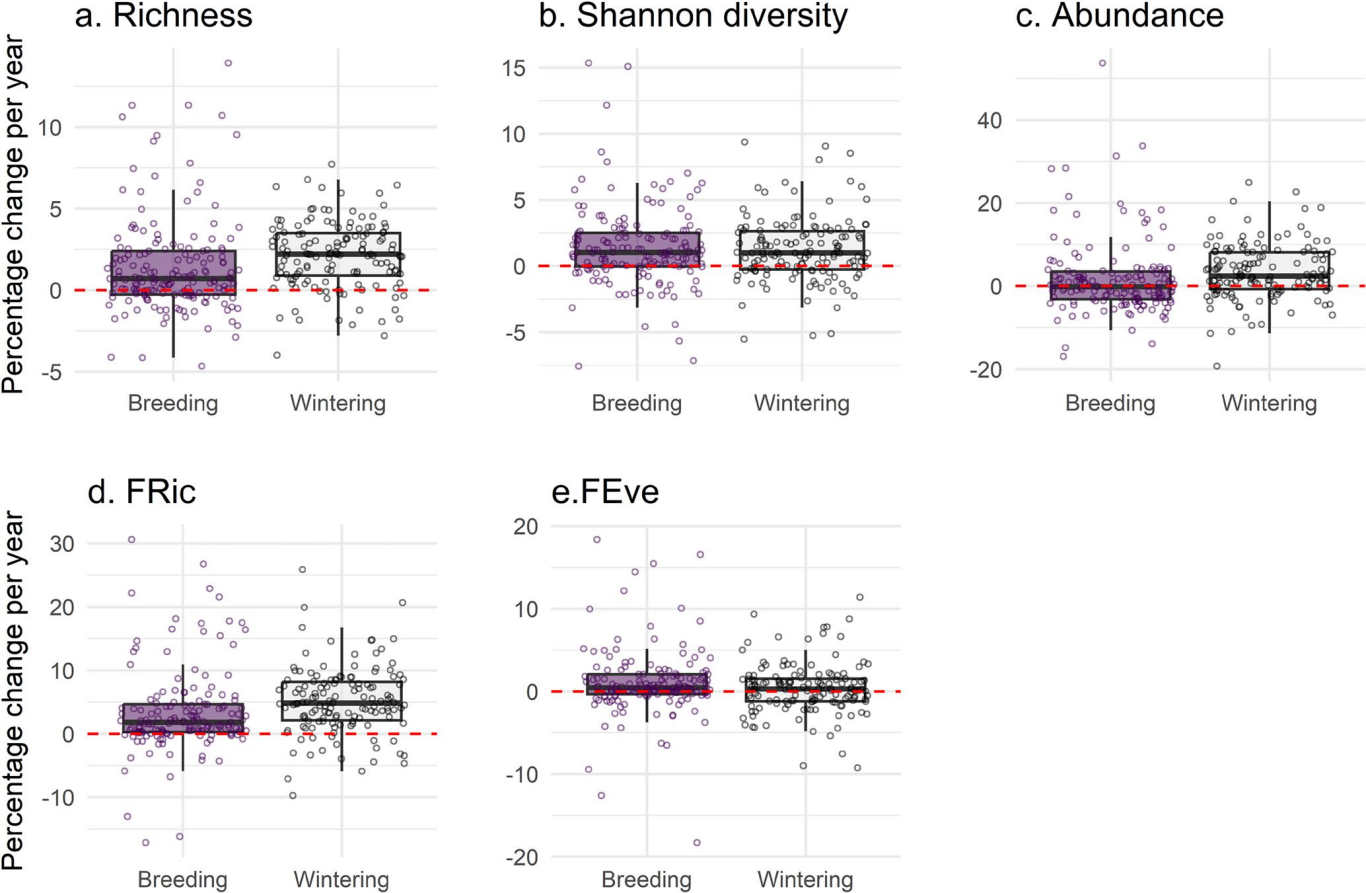
Site‐level trends by sampling season for (a) taxonomic richness, (b) Shannon diversity, (c) abundance, (d) functional richness (FRic) and (e) functional evenness (FEve) across 291 sites in the Baltic Sea (*n*_Breeding_ = 153; *n*_Wintering_ = 138). Circles show site values. Boxes correspond to the 25th and 75th percentiles; lines inside a box show the median; whiskers extend to the last observation within 1.5 times the interquartile range from the quartiles. The horizontal dashed red line shows the location of no change.

Overall, wintering bird communities in the Baltic Sea exhibited a faster recovery than breeding communities (Figure 5). More specifically, increments in taxonomic (*R* ^2^ = 0.019; *p* = 0.019) and functional richness (*R* ^2^ = 0.020; *p* = 0.016) were more pronounced in wintering than in breeding communities. In contrast, we did not find significant differences in Shannon diversity (*R* ^2^ < 0.001; *p* = 0.647), abundance (*R* ^2^ = 0.011; *p* = 0.077) and functional diversity trends (*R* ^2^ = 0.008; *p* = 0.132) between the two sampling seasons. Additionally, the dispersion of trends for all metrics was homogeneous between the two sampling seasons.

## 4 Discussion

Using a comprehensive dataset of 308 time series covering a wide latitudinal and climatic gradient in Europe, we document the recovery of coastal bird communities over 68 years (1957 to 2024) in the Baltic Sea, the Greater North Sea and the Western Mediterranean Sea. The observed increases in abundance and taxonomic diversity are mirrored by increases in functional diversity, supporting a broad recovery process. However, caution is needed in assuming that the observed recoveries in coastal bird communities will necessarily continue. In fact, our results showed that recovery trends in the Western Mediterranean have stagnated since the early 2000s. In addition, we found a high variability in overall trends across sites, including 4.5% of sites showing significant declines in species richness, 5.2% of sites showing declines in Shannon diversity and 13.6% of sites showing declines in abundance, reflecting local differences in community trends. Many bird populations and communities are still threatened in Europe by habitat loss and sea use changes (Busch et al. 2013), overfishing (Bastardie et al. 2024), pollutants (Colomer‐Vidal et al. 2022) and the introduction of invasive species (Katsanevakis et al. 2023). Our results emphasize the relevance of continuing to reduce broader environmental pressures on coastal bird communities and underscore the importance of sustaining and enhancing current conservation measures that have proven effective.

Observed recovery trends in coastal bird communities are consistent with documented recolonization of different marine birds and increments in breeding success after local restoration measures in Europe, contributing to higher taxonomic richness, greater community diversity and increased overall bird abundance (Bried et al. 2009; Le Corre et al. 2015). Similarly, Grandgeorge et al. (2008) showed that the abundance of marine birds in Britain and Ireland increased at an average rate of 1% per year between 1969 and 2002, largely due to long‐standing protection measures that reduced their exploitation and persecution. Some extreme cases of population recovery in Europe have also been reported, such as the Audouin's Gull ( *Ichthyaetus audouinii* ) colony in the Ebro Delta (Spain), which grew exponentially at rates exceeding 40% per year between 1980 and 2000 (Oro and Ruxton 2001) before collapsing due to the arrival of invasive carnivores (Oro et al. 2023). Observed community recoveries in our study could potentially have been driven by different causes including natural recolonization of habitats from which species had been extirpated (Lotze 2005), the formation of new colonies at suitable sites (Oro and Ruxton 2001), assisted reintroductions (O'Rourke 2014; Spatz et al. 2023), the eradication of predators from islands, islets and coastal habitats (Capizzi et al. 2016; Le Corre et al. 2015), and the implementation of protection measures and environmental legislation (Grandgeorge et al. 2008).

Our results also align with those reported for other ecosystems and taxonomic groups (Ledger et al. 2022). On a global scale, 42% of marine mammal species have exhibited significant population increases in recent decades, particularly pinnipeds, sirenians, polar bears and otters, with only 10% of species experiencing declines (Magera et al. 2013). Similarly, riverine macroinvertebrate communities in Europe have experienced increases in taxon richness (0.7% per year), functional richness (2.4% per year), and abundance (1.2% per year), although these trends primarily occurred before the 2010s and have since stagnated (Haase et al. 2023; Sinclair et al. 2024). The overarching drivers of these positive trends in Europe include the implementation of stronger conservation policies, such as the Marine Strategy Framework Directive (MSFD) and the Water Framework Directive (WFD), coupled with land‐use de‐intensification and growing public support for conservation efforts (Chapron et al. 2014; Davoli et al. 2024; Haase et al. 2023).

### 4.1 The Effect of Protected Areas

Our results show that strictly protected areas (i.e., IUCN Categories Ia, Ib, and II) in comparison to less strictly protected sites support the conservation and recovery of coastal bird communities. While more strictly protected sites demonstrated average annual increases in taxonomic richness, Shannon diversity and functional evenness of 2.1 to 3.1% during the study period, and nonprotected sites increases of 0.6 to 1.6%; less protected sites (i.e., IUCN Categories of Protected Areas III, IV, V, VI, or other kinds of protection) exhibited more modest increases or even slight declines. This result aligns with a previous study on forest bird species, which found that strict protection is strongly associated with higher bird occurrence (Timmers et al. 2022). It also reinforces the importance of conservation efforts made to restore and maintain bird populations to achieve a good conservation status, motivated by the European legislation (e.g., EU Bird Directive), and more specifically by the development of European Bird Species Action Plans for threatened birds such as the Balearic shearwater ( *Puffinus mauretanicus* ), Audouin's gull, the European shag (*Gulosus aristotelis*) or the red‐breasted goose ( *Branta ruficollis* ) (BirdLife International 2011).

However, our results also showed that nonprotected sites have also recovered their coastal bird communities during the last decades at a rate similar to that of strictly protected areas. For example, the two nonprotected sites studied in the Western Mediterranean Sea, which are located in the Port of Valencia and the Port of Castellón, showed high average percentages of change in taxonomic richness (3.4% per year), Shannon diversity (5.1%) and especially abundance (37.9% per year). Species such as Adouin's gull and the Sandwich tern ( *Sterna sandvicensis* ) have successfully established themselves in these ports over the past few decades due to restricted access, fencing and the general absence of terrestrial predators (Payo‐Payo et al. 2017). Similarly, the 40 nonprotected sites in the Baltic Sea—including sites adjacent to anthropogenic areas such as the Port of Tallinn, Muuga Harbour and Pärnu Bay—exhibited rates of change comparable to those observed in strictly protected areas. These results may be consistent with the recovery of ecosystems within estuaries, surrounding urban coastal areas and other nonprotected coastal sites over the last decades, which were highly polluted and degraded environments in the recent past (Haase et al. 2023; Pascual et al. 2012). Efforts to improve these areas, including the implementation of stricter environmental regulations, hunting prohibitions, industrial improvements and urban renewal projects, have likely contributed to the restoration of nonprotected habitats and the recovery of their coastal bird populations (Airoldi et al. 2021; Sarzo et al. 2011). In addition, many species demonstrate broad ecological plasticity, allowing them to adapt to human‐altered environments—especially in the absence of direct persecution—which may further explain their successful establishment and recovery in nonprotected areas (Martínez‐Abraín et al. 2019a, 2019b). Altogether, these results highlight the complex and dynamic habitat use patterns of coastal birds, shaped by a combination of habitat protection, policy improvements, shifts in human activity, and other environmental factors; and they may also point to possible spillover benefits from adjacent large‐scale marine protected areas (Lynham and Villaseñor‐Derbez 2024).

### 4.2 Contrasting Trends Between Wintering and Breeding Communities

Our results indicate that wintering bird communities in the Baltic Sea have shown a faster recovery than breeding communities in terms of both taxonomic and functional richness. This faster recovery of wintering bird communities may have been driven by the significant reduction (54.7%) in bird bycatch in the Baltic Sea between the 1970s and 2010s (Marchowski 2022). In fact, bird bycatch has long been a major threat to wintering bird populations in this region (Stempniewicz 1994). Additionally, climate change—particularly the warming of winter temperatures—may be driving shifts in both the wintering and breeding ranges of certain bird species. In the case of the Baltic Sea, milder winters may have reduced the need for long‐distance migration in some species, thereby contributing to the observed increases in species richness and diversity within coastal wintering bird communities (Frederiksen et al. 2018; Pavón‐Jordán et al. 2015; Potvin et al. 2016). For instance, the Great Cormorant is increasingly wintering along the coasts of Estonia, with its populations experiencing significant growth in recent decades (Frederiksen et al. 2018).

However, previous studies have witnessed a significant drop in some wintering seabird populations in the Baltic Sea in recent decades because of oil pollution due to ship traffic, and bird mortality due to bycatch, hunting and offshore wind farms (Skov et al. 2011). For instance, the long‐tailed duck, the most numerous waterbird in the region, suffered a decline of 65% from 1992–1993 to 2007–2009 (Skov et al. 2011). Similarly, common eider decreased by 51%, common scoter ( *Melanitta nigra* ) by 47% and red‐breasted Merganser ( *Mergus serrator* ) by 42% during the same period (Skov et al. 2011). These observations partially align with our results, which indicate a phase of decline in Baltic bird communities between the 1980s and 2000s, followed by a more pronounced recovery of the communities.

### 4.3 Research Limitations and Future Directions

Although our results are supported by a robust dataset covering a broad latitudinal gradient and align with previous studies evaluating changes in marine bird communities in Europe over the last decades (Grandgeorge et al. 2008), they are limited by various aspects. First, our study is spatially restricted to only coastal sites for which we obtained data that met the established requirements. The Baltic Sea coast of Estonia, which has the highest number of time series, and the Mediterranean coast of Spain, are both well‐represented with a uniform distribution of sampling points across each region. However, the Greater North Sea has very limited representation, with only two time series meeting the selection criteria. Consequently, findings from this area may not accurately capture broader regional trends. Additionally, the inclusion of more time series from the Greater North Sea and the Western Mediterranean Sea would likely reveal more marked regional differences in biodiversity trends that could not be observed in this study due to the limited data available from certain areas. Another important ecological factor is the nomadic behavior of some species, such as Audouin's gulls, which complicates effective monitoring at both regional and transnational scales (Martínez‐Abraín et al. 2003).

Second, our analyses were temporally restricted, especially when evaluating the effect of protected areas, as monitoring data was primarily available from the early 1990s onward (see also Cano‐Barbacil et al. 2025), meaning we could not compare changes before and after protection designation (but see Martínez‐Abraín et al. 2016). As a result, our findings primarily capture changes in bird communities over the past three decades after sites became protected. This means we may be missing major recoveries that occurred earlier, as the first laws and regulations prohibiting hunting and the collection of eggs and chicks in Europe date back to the late 19th and early 20th centuries (e.g., Sea Birds Preservation Act 1869 in the United Kingdom), even before the designation of most protected areas (Ferrero‐García 2013, 2020). In addition, birds often exhibit slow life cycles and population dynamics, with generation times that can span many years. Thus, the use of relatively short time series (15% of our time series cover less than 20‐year periods) may only capture a small portion of these changes, missing delayed responses to environmental disturbances.

Finally, we were only able to analyse the trends of breeding and wintering bird communities separately in the Baltic Sea due to a lack of data from the other regions studied. However, as our results indicate, separating the analysis of these two communities could provide deeper insights into the specific responses of migratory and resident birds, which might otherwise be obscured.

Future studies would greatly benefit from the inclusion of data covering a broader range of regional seas across Europe to obtain more representative trends at a continental scale. Achieving this requires improving data accessibility, as substantial datasets on bird populations already exist—birds being among the most intensively studied and monitored organisms globally—yet many of these are not openly available for research purposes (Rodríguez‐Caro et al. 2024). For instance, within the EMODnet database, the dataset from Estonia was the only publicly available one that met our requirements. Increasing the availability of such datasets through open data initiatives and collaborative platforms (e.g., BioTIME; Dornelas et al. 2018, 2025) would enhance the capacity to conduct comprehensive analyses and improve our understanding of large‐scale biodiversity trends.

### 4.4 Concluding Remarks

Long‐term and large‐scale studies evaluating biodiversity trends remain relatively scarce, highlighting significant gaps in our understanding of biodiversity changes and their interactions with human‐induced pressures. Although overall positive trends have been observed, local population declines persist in various regions. For improving sites, future changes will depend on the site‐specific recovery phase achieved so far. In some sites, recovery may be complete, resulting in a stationary trend, while in other sites, remaining stressors may have stalled the positive trend. Moreover, broader environmental pressures including overfishing, bycatch mortality in fisheries, habitat degradation, pollution and climate change, continue to pose serious threats to coastal and marine ecosystems. Addressing these challenges requires not only the establishment of strictly protected areas but also their effective management to ensure long‐term conservation success. Strengthening monitoring programs, fostering international collaboration and integrating adaptive conservation strategies will be crucial steps toward mitigating biodiversity loss and promoting resilience in European seas.

## Author Contributions

**Carlos Cano‐Barbacil:** conceptualization, data curation, formal analysis, investigation, methodology, writing – original draft. **Diana E. Bowler:** formal analysis, methodology, writing – review and editing. **Gustavo A. Ballesteros‐Pelegrín:** data curation, writing – review and editing. **Albert Bertolero:** data curation, writing – review and editing. **Klaas Deneudt:** data curation. **Meritxell Genovart:** data curation, writing – review and editing. **Miguel Ángel Gómez‐Serrano:** data curation, writing – review and editing. **Antonio J. Hernández‐Navarro:** data curation, writing – review and editing. **Daniel Oro:** data curation, writing – review and editing. **Antonio Zamora‐López:** data curation, writing – review and editing. **Peter Haase:** conceptualization, funding acquisition, writing – review and editing.

## Funding

This study was funded by the European Union under the Horizon Europe Programme, Grant Agreement No. 101082021 (MARCO‐BOLO). Additional funding for authors was provided by the European Union Horizon 2020 project eLTER PLUS (grant number 871128). Views and opinions expressed are however those of the author(s) only and do not necessarily reflect those of the European Union or European Research Executive Agency (REA). Neither the European Union nor the granting authority can be held responsible for them. C.C.‐B. benefited from a postdoctoral contract (ref. JDC2023‐052358‐I), funded by the Spanish Ministry of Science, Innovation, and Universities (MICIU/AEI/10.13039/501100011033) and the FSE+. Monitoring of Western Mediterranean Sea marine birds was funded by the Spanish Ministry of Science and EU FEDER funds with several grants (ref. PID2021‐122893NB‐C21, PID2021‐124731NB‐I00).

## Conflicts of Interest

The authors declare no conflicts of interest.

## Supporting information


**Table S1:** List of bird species recorded in each region and season.
**Table S2:** Description of the seven datasets used in this study, including details on the data source, the region studied, the number of time series meeting the established criteria, and the sampling protocol applied.
**Figure S1:**. Distribution of time series by the number of sampling campaigns conducted.
**Figure S2:**. Distribution of total observations by year.
**Table S3:**. List of 13 morphological and life‐history bird traits collected and used to calculate functional richness and evenness.
**Figure S3:**. Marine and coastal protected areas in (a) the Baltic Sea (Estonia) and (b) the Western Mediterranean Sea (Spain).
**Table S4:**. Definition of the different IUCN protected area categories.
**Table S5:**. Equation used to calculate the percentage of change per year.
**Figure S4:**. Site‐level trends expressed as percentage of change per year for (a) taxonomic richness, (b) taxonomic diversity (i.e., Shannon diversity index), (c) total abundance, (d) functional richness (FRic), and (e) functional evenness (FEve) across 308 sites.
**Figure S5:** Abundance trend estimates for species sampled in the Baltic Sea.
**Figure S6:** Temporal changes in abundance for the nine most common species in the Baltic Sea, based on generalized additive mixed models. Each line represents the trend at a single sampling site.
**Figure S7:** Abundance trend estimates for species sampled in the Greater North Sea.
**Figure S8:** Temporal changes in abundance for the nine most common species in the Greater North Sea, based on generalized additive mixed models. Each line represents the trend at a single sampling site.
**Figure S9:** Abundance trend estimates for species sampled in the Western Mediterranean Sea.
**Figure S10:** Temporal changes in abundance for the nine most common species in the Western Mediterranean Sea, based on generalized additive mixed models. Each line represents the trend at a single sampling site.
**Appendix S1:** Meta‐analytic multivariate model results.
**Table S6:**. Estimate percentage of change in biodiversity metrics for the Baltic Sea (BS), the Greater North Sea (GNS) and the Western Mediterranean Sea (WMS). Estimates significantly different from zero are highlighted in bold (*p ≤ 0.05; **p ≤ 0.01; ***p ≤ 0.001).
**Table S7:** Results of the linear mixed models.

## Data Availability

The data and code supporting the trend analysis are available on Figshare (https://doi.org/10.6084/m9.figshare.29314334.v1).
