# Supplementary material for: Almost Seven Decades of Coastal Bird Community Recovery Across Three European Seas
Source: Glob Chang Biol. 2025 Nov 26;31(11):e70623. doi: 10.1111/gcb.70623 (PMC12648369; doi:10.1111/gcb.70623)
Supplement: Supplementary file 1 — Table S1: List of bird species recorded in each region and season. Table S2: Description of the seven datasets used in this study, including details on the data source, the region studied, the number of time series meeting the established criteria, and the sampling protocol applied. Figure S1:. Distribution of time series by the number of sampling campaigns conducted. Figure S2:. Distribution of total observations by year. Table S3:. List of 13 morphological and life‐history bird traits collected and used to calculate functional richness and evenness. Figure S3:. Marine and coastal protected areas in (a) the Baltic Sea (Estonia) and (b) the Western Mediterranean Sea (Spain). Table S4:. Definition of the different IUCN protected area categories. Table S5:. Equation used to calculate the percentage of change per year. Figure S4:. Site‐level trends expressed as percentage of change per year for (a) taxonomic richness, (b) taxonomic diversity (i.e., Shannon diversity index), (c) total abundance, (d) functional richness (FRic), and (e) functional evenness (FEve) across 308 sites. Figure S5: Abundance trend estimates for species sampled in the Baltic Sea. Figure S6: Temporal changes in abundance for the nine most common species in the Baltic Sea, based on generalized additive mixed models. Each line represents the trend at a single sampling site. Figure S7: Abundance trend estimates for species sampled in the Greater North Sea. Figure S8: Temporal changes in abundance for the nine most common species in the Greater North Sea, based on generalized additive mixed models. Each line represents the trend at a single sampling site. Figure S9: Abundance trend estimates for species sampled in the Western Mediterranean Sea. Figure S10: Temporal changes in abundance for the nine most common species in the Western Mediterranean Sea, based on generalized additive mixed models. Each line represents the trend at a single sampling site. Appendix S1: Meta‐analytic multivariate model resu [file GCB-31-e70623-s001.docx]

**Supplementary material: Almost seven decades of coastal bird community recovery across three European seas**

Carlos Cano-Barbacil^1,2^*; Diana E. Bowler^3^; Gustavo A. Ballesteros-Pelegrín^4,5^; Albert Bertolero^6^; Klaas Deneudt^7^; Meritxell Genovart^8^; Miguel Ángel Gómez-Serrano^9^; Antonio J. Hernández-Navarro^4^; Daniel Oro^8^; Antonio Zamora-López^4,10^; Peter Haase^1,11^

^1^ Senckenberg Research Institute and Natural History Museum Frankfurt, Department of River Ecology and Conservation, Gelnhausen, Germany

^2^ Departamento de Biodiversidad y Biología Evolutiva, Museo Nacional de Ciencias Naturales, CSIC, Madrid 28006, Spain

^3^ Biodiversity Monitoring & Analysis, UK Centre for Ecology & Hydrology, Wallingford, UK

^4^ Autonomous University of Madrid. Faculty of Philosophy and Letters, Department of Geography

^5^ ANSE-Association of Naturalists of the Southeast

^6^ Associació Ornitològica Picampall de les Terres de l'Ebre. Amposta, Spain.

^7^ Flanders Marine Institute (VLIZ) InnovOcean Campus, Jacobsenstraat 1, 8400 Ostend, Belgium

^8^ CEAB (CSIC), Department of Ecology and Complexity, Theoretical and Computational Ecology Group, Blanes, Catalonia, Spain

^9^ Cavanilles Institute of Biodiversity and Evolutionary Biology, University of Valencia. Spain.

^10^ University of Murcia. Faculty of Biology, Department of Zoology and Physical Anthropology

^11^ University of Duisburg-Essen, Faculty of Biology, Essen, Germany

*Corresponding author: Carlos Cano-Barbacil Address: Museo Nacional de Ciencias Naturales, C/ José Gutiérrez Abascal, 2, 28006 Madrid, Spain. Email: [carlos.cano@mncn.csic.es](mailto:carlos.cano@mncn.csic.es)

**Table S1.** List of bird species recorded in each region and season. Species are also classified according to their migratory status (sedentary, short-distance migrant and long-distant migrant). Bs = Breeding season; Ws = Wintering season.

| **Species** | **Family** | **Baltic Sea** | **Greater North Sea** | **Western Mediterranean Sea** | **Sedentary** | **Short-distance migrant** | **Long-distance migrant** |
| --- | --- | --- | --- | --- | --- | --- | --- |
| *Actitis hypoleucos* | Scolopacidae | Bs |  | Ws | 0 | 0 | 1 |
| *Alca torda* | Alcidae | Bs, Ws |  | Ws | 0 | 1 | 0 |
| *Alcedo atthis* | Alcedinidae |  |  | Ws | 1 | 1 | 0 |
| *Alopochen aegyptiaca* | Anatidae |  | Ws |  | 1 | 0 | 0 |
| *Anas acuta* | Anatidae | Bs | Ws | Ws | 0 | 1 | 0 |
| *Anas crecca* | Anatidae | Bs, Ws | Ws |  | 0 | 1 | 0 |
| *Anas platyrhynchos* | Anatidae | Bs, Ws | Ws | Ws | 0 | 1 | 0 |
| *Anas strepera* | Anatidae | Bs, Ws | Ws | Ws | 1 | 1 | 0 |
| *Anser albifrons* | Anatidae |  | Ws |  | 0 | 1 | 0 |
| *Anser anser* | Anatidae | Bs, Ws | Ws | Ws | 0 | 1 | 0 |
| *Anser brachyrhynchus* | Anatidae |  | Ws |  | 0 | 1 | 0 |
| *Anser caerulescens* | Anatidae |  | Ws |  | 0 | 1 | 0 |
| *Anser fabalis* | Anatidae |  | Ws |  | 0 | 1 | 0 |
| *Anser serrirostris* | Anatidae |  | Ws |  | 0 | 1 | 0 |
| *Ardea alba* | Ardeidae |  | Ws | Ws | 0 | 1 | 0 |
| *Ardea cinerea* | Ardeidae |  | Ws | Ws | 0 | 1 | 0 |
| *Arenaria interpres* | Scolopacidae | Bs | Ws | Ws | 0 | 1 | 0 |
| *Aythya ferina* | Anatidae | Bs, Ws | Ws |  | 0 | 1 | 0 |
| *Aythya fuligula* | Anatidae | Bs, Ws | Ws |  | 0 | 1 | 0 |
| *Aythya marila* | Anatidae | Bs, Ws | Ws |  | 0 | 1 | 0 |
| *Branta bernicla* | Anatidae |  | Ws |  | 0 | 1 | 0 |
| *Branta canadensis* | Anatidae | Bs, Ws | Ws |  | 1 | 1 | 0 |
| *Branta hutchinsii* | Anatidae |  | Ws |  | 1 | 1 | 0 |
| *Branta leucopsis* | Anatidae | Bs, Ws | Ws |  | 0 | 1 | 0 |
| *Branta ruficollis* | Anatidae |  | Ws |  | 1 | 1 | 0 |
| *Bucephala clangula* | Anatidae | Bs, Ws | Ws |  | 0 | 1 | 0 |
| *Calidris alba* | Scolopacidae |  | Ws |  | 0 | 0 | 1 |
| *Calidris alpina* | Scolopacidae | Bs | Ws | Ws | 0 | 1 | 0 |
| *Calidris canutus* | Scolopacidae |  | Ws |  | 0 | 0 | 1 |
| *Calidris ferruginea* | Scolopacidae |  | Ws |  | 0 | 1 | 1 |
| *Calidris maritima* | Scolopacidae | Ws | Ws |  | 0 | 1 | 0 |
| *Calidris minuta* | Scolopacidae |  | Ws |  | 0 | 0 | 1 |
| *Calonectris diomedea* | Procellariidae |  |  | Bs, Ws | 0 | 0 | 1 |
| *Cepphus grylle* | Alcidae | Ws |  |  | 1 | 0 | 0 |
| *Charadrius alexandrinus* | Charadriidae |  | Ws | Bs, Ws | 0 | 1 | 1 |
| *Charadrius dubius* | Charadriidae | Bs |  | Bs | 0 | 0 | 1 |
| *Charadrius hiaticula* | Charadriidae | Bs | Ws | Ws | 0 | 1 | 1 |
| *Chlidonias hybrida* | Laridae |  |  | Bs | 0 | 0 | 1 |
| *Clangula hyemalis* | Anatidae | Ws | Ws | Ws | 0 | 1 | 0 |
| *Cygnus columbianus* | Anatidae | Ws | Ws |  | 0 | 1 | 0 |
| *Cygnus cygnus* | Anatidae | Ws | Ws |  | 0 | 1 | 0 |
| *Cygnus olor* | Anatidae | Bs, Ws | Ws | Ws | 1 | 1 | 0 |
| *Egretta garzetta* | Ardeidae |  | Ws | Ws | 0 | 0 | 1 |
| *Fulica atra* | Rallidae |  | Ws | Ws | 1 | 1 | 0 |
| *Gallinago gallinago* | Scolopacidae |  | Ws |  | 0 | 1 | 1 |
| *Gallinula chloropus* | Rallidae |  | Ws | Ws | 1 | 1 | 0 |
| *Gavia arctica* | Gaviidae | Ws |  |  | 0 | 1 | 0 |
| *Gavia stellata* | Gaviidae | Ws |  |  | 0 | 1 | 0 |
| *Gelochelidon nilotica nilotica* | Laridae |  |  | Bs | 0 | 0 | 1 |
| *Glareola pratincola* | Glareolidae |  |  | Bs | 0 | 0 | 1 |
| *Grus grus* | Gruidae | Bs |  |  | 0 | 0 | 1 |
| *Gulosus aristotelis* | Phalacrocoracidae |  |  | Bs, Ws | 1 | 0 | 0 |
| *Haematopus ostralegus* | Charadriidae | Bs | Ws | Ws | 0 | 1 | 0 |
| *Haliaeetus albicilla* | Accipitridae | Bs, Ws |  |  | 1 | 0 | 0 |
| *Himantopus himantopus* | Recurvirostridae |  |  | Ws | 0 | 0 | 1 |
| *Hydrocoloeus minutus* | Laridae | Bs, Ws | Ws |  | 0 | 1 | 0 |
| *Hydroprogne caspia* | Laridae | Bs |  |  | 0 | 0 | 1 |
| *Ichthyaetus melanocephalus* | Laridae |  | Ws | Bs, Ws | 0 | 1 | 0 |
| *Larus argentatus* | Laridae | Bs, Ws | Ws |  | 0 | 1 | 0 |
| *Larus audouinii* | Laridae |  |  | Bs, Ws | 0 | 1 | 0 |
| *Larus canus* | Laridae | Bs, Ws | Ws |  | 0 | 1 | 0 |
| *Larus fuscus* | Laridae | Bs |  | Bs, Ws | 0 | 1 | 0 |
| *Larus genei* | Laridae |  |  | Bs, Ws | 0 | 1 | 0 |
| *Larus glaucoides* | Laridae |  | Ws |  | 0 | 1 | 0 |
| *Larus hyperboreus* | Laridae |  | Ws |  | 0 | 1 | 0 |
| *Larus marinus* | Laridae | Bs, Ws | Ws |  | 0 | 1 | 0 |
| *Larus michahellis* | Laridae |  |  | Bs | 0 | 1 | 0 |
| *Larus ridibundus* | Laridae | Bs, Ws | Ws | Bs, Ws | 0 | 1 | 0 |
| *Limosa lapponica* | Scolopacidae |  | Ws |  | 0 | 1 | 0 |
| *Limosa limosa* | Scolopacidae |  | Ws | Ws | 0 | 0 | 1 |
| *Lophodytes cucullatus* | Anatidae | Ws |  |  | 0 | 1 | 0 |
| *Lymnocryptes minimus* | Scolopacidae |  | Ws |  | 0 | 1 | 1 |
| *Mareca penelope* | Anatidae | Bs, Ws | Ws | Ws | 0 | 1 | 0 |
| *Marmaronetta angustirostris* | Anatidae |  |  | Ws | 0 | 1 | 0 |
| *Melanitta fusca* | Anatidae | Bs, Ws | Ws | Ws | 0 | 1 | 0 |
| *Melanitta nigra* | Anatidae | Ws | Ws | Ws | 0 | 1 | 0 |
| *Mergellus albellus* | Anatidae | Ws | Ws |  | 0 | 1 | 0 |
| *Mergus merganser* | Anatidae | Bs, Ws | Ws | Ws | 0 | 1 | 0 |
| *Mergus serrator* | Anatidae | Bs, Ws | Ws | Ws | 0 | 1 | 0 |
| *Netta rufina* | Anatidae |  |  | Ws | 0 | 1 | 0 |
| *Numenius arquata* | Scolopacidae | Bs | Ws | Ws | 0 | 1 | 1 |
| *Numenius phaeopus* | Scolopacidae | Bs | Ws | Ws | 0 | 0 | 1 |
| *Pandion haliaetus* | Pandionidae |  |  | Ws | 0 | 0 | 1 |
| *Panurus biarmicus* | Sylviidae | Bs, Ws |  |  | 1 | 0 | 0 |
| *Phalacrocorax carbo* | Phalacrocoracidae | Bs, Ws | Ws | Ws | 0 | 1 | 0 |
| *Philomachus pugnax* | Scolopacidae | Bs | Ws |  | 0 | 0 | 1 |
| *Phoenicopterus ruber* | Phoenicopteridae |  |  | Ws | 0 | 1 | 0 |
| *Platalea leucorodia* | Threskiornithidae |  | Ws | Ws | 0 | 1 | 0 |
| *Pluvialis apricaria* | Charadriidae |  | Ws |  | 0 | 1 | 0 |
| *Pluvialis squatarola* | Charadriidae |  | Ws | Ws | 0 | 1 | 1 |
| *Podiceps auritus* | Podicipedidae | Bs, Ws |  |  | 0 | 1 | 0 |
| *Podiceps cristatus* | Podicipedidae | Bs, Ws | Ws | Ws | 0 | 1 | 0 |
| *Podiceps grisegena* | Podicipedidae | Ws |  |  | 0 | 1 | 0 |
| *Podiceps nigricollis* | Podicipedidae |  |  | Ws | 0 | 1 | 0 |
| *Polysticta stelleri* | Anatidae | Ws |  |  | 0 | 1 | 0 |
| *Rallus aquaticus* | Rallidae | Ws | Ws |  | 1 | 1 | 0 |
| *Recurvirostra avosetta* | Recurvirostridae | Bs | Ws | Ws | 0 | 1 | 0 |
| *Rissa tridactyla* | Laridae |  | Ws |  | 1 | 0 | 0 |
| *Scolopax rusticola* | Scolopacidae |  | Ws |  | 0 | 1 | 0 |
| *Somateria mollissima* | Anatidae | Bs, Ws | Ws | Ws | 0 | 1 | 0 |
| *Spatula clypeata* | Anatidae | Bs | Ws | Ws | 0 | 1 | 0 |
| *Spatula querquedula* | Anatidae | Bs |  |  | 0 | 0 | 1 |
| *Sterna hirundo* | Sternidae | Bs | Ws | Bs, Ws | 0 | 0 | 1 |
| *Sterna paradisaea* | Sternidae | Bs |  |  | 0 | 0 | 1 |
| *Sterna sandvicensis* | Sternidae | Bs | Ws | Bs, Ws | 0 | 0 | 1 |
| *Sternula albifrons* | Sternidae | Bs |  | Bs | 0 | 1 | 0 |
| *Tachybaptus ruficollis* | Podicipedidae | Ws | Ws | Ws | 0 | 1 | 0 |
| *Tadorna tadorna* | Anatidae | Bs | Ws | Ws | 0 | 1 | 0 |
| *Tringa erythropus* | Scolopacidae |  | Ws |  | 0 | 0 | 1 |
| *Tringa nebularia* | Scolopacidae |  | Ws | Ws | 0 | 0 | 1 |
| *Tringa ochropus* | Scolopacidae |  | Ws |  | 0 | 1 | 1 |
| *Tringa totanus* | Scolopacidae | Bs | Ws | Ws | 0 | 1 | 1 |
| *Uria aalge* | Alcidae | Ws |  |  | 1 | 0 | 0 |
| *Vanellus vanellus* | Charadriidae |  | Ws |  | 0 | 1 | 0 |

**Table S2.** Description of the seven datasets used in this study, including details on the data source, the region studied, the number of time series meeting the established criteria, and the sampling protocol applied.

| **Source** | **Dataset ID** | **Sea**  **(Country)** | ***n* time series** | **Sampling protocol** |
| --- | --- | --- | --- | --- |
| EMODnet | emodnet_6026 | Baltic Sea (Estonia) | 153 | Visual census to record counts of breeding birds on small sea islands and islets within Estonian territorial waters, as part of a national monitoring program. Observers followed free-choice routes, conducting point counts. Each route includes 20 observation points, where a 5-minute count is conducted at each stop. To minimize double-counting, points are spaced at least 200 meters apart in closed landscapes and 300 meters apart in open landscapes. |
| EMODnet | emodnet_6027 | Baltic Sea (Estonia) | 138 | Mid-winter visual census to record individual counts of waterfowl species. Observers scanned flocks of waterbirds, typically consisting of multiple species, using either a telescope or binoculars depending on visibility and distance. Counts are conducted by recording each species individually or by estimating in larger groups (“blocks”) for higher numbers. |
| Pilotto et al. (2020) | lter_be | Greater North Sea (Belgium) | 1 | The bird abundance data is based on counts of individuals present in tidal marshes during low tide. Counts are conducted from a boat, covering a soft substrate tidal flat area of 385 hectares. |
| Pilotto et al. (2020) | lter_nl | Greater North Sea (The Netherlands) | 1 | Bird density (number of individuals per km²) is determined through counts conducted during high tide, when non-breeding birds roost on land, while two duck species that remain on the water are counted separately from an airplane. |
| Data call | dc_es_ebro | Western Mediterranean Sea (Spain) | 1 | Visual censuses were conducted over 1 to 4 days, depending on the colony's size, during the second week of May, when most pairs had laid eggs, but few broods had hatched. A team of four to twelve people, adjusted according to the size and layout of the main colony and its sub-colonies, counted the nests. |
| Data call | dc_es_mmenor | Western Mediterranean Sea (Spain) | 1 | Surveys were consistently conducted during the second week of January. A team of at least three people performs visual censuses from a boat, following a standardized route throughout the Mar Menor lagoon, covering its entire area (135.76 km²) and pausing as needed to count waterbirds. Using binoculars, a telescope, and a camera, the team recorded the total number of individuals for each species observed either on the water or along the shores of the islands. |

| **Source** | **Dataset** | **Sea (Country)** | ***n* time series** | **Sampling method** |
| --- | --- | --- | --- | --- |
| Data call | dc_es_med | Western Mediterranean Sea (Spain) | 13 | During each species' breeding season, all pairs present in breeding colonies and all occupied nests were counted. Surveys covered all breeding habitats at each location, including cliffs, beaches, islands, salt marshes, marshes, and coastal lagoons. Census methods remained consistent across all years and species, ensuring standardized data collection throughout the monitoring period. The data primarily originate from monitoring programs for marine and coastal bird species in the Valencian Community, coordinated regionally by the Generalitat Valenciana. |


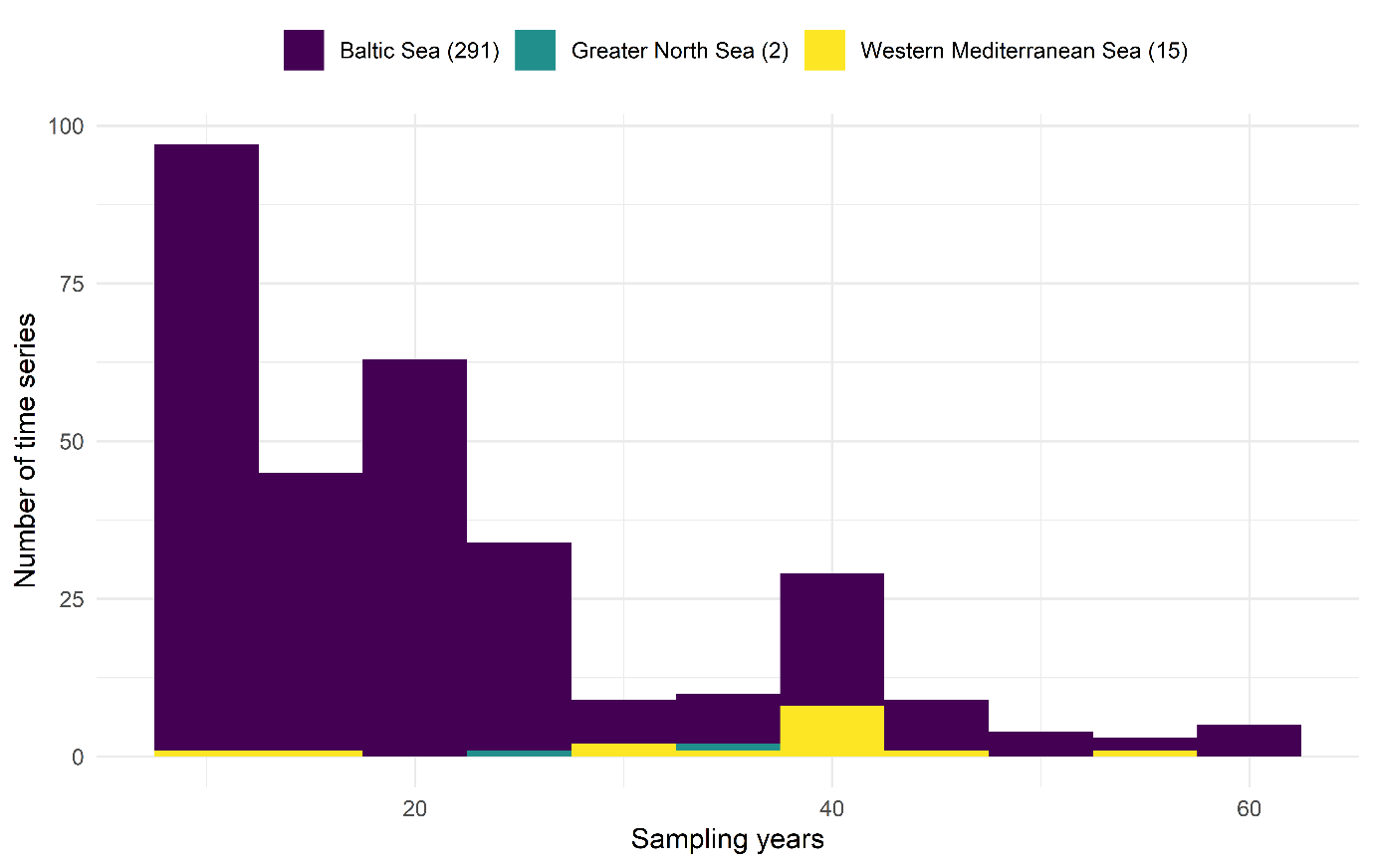


**Figure S1.** Distribution of time series by the number of sampling campaigns conducted. The total number of time series for each regional sea is indicated in brackets.


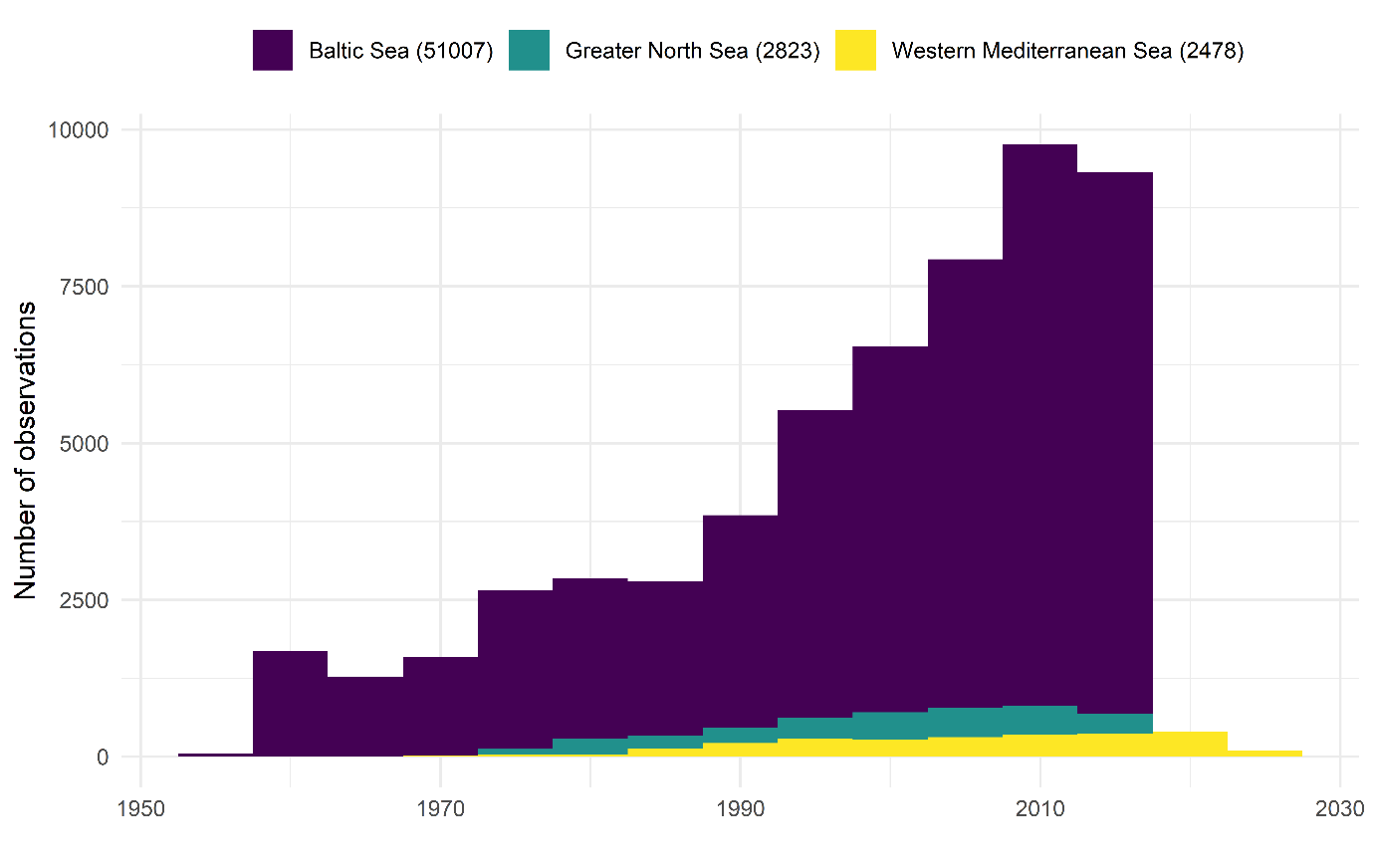


**Figure S2.** Distribution of total observations by year. The total number of observations for each regional sea is indicated in brackets.

**Table S3.** List of 13 morphological and life-history bird traits collected and used to calculate functional richness and evenness.

| **Trait** | **Definition** |
| --- | --- |
| Length | Mean length of the bird, unsexed (cm) |
| Wing length | Mean length of the wing, unsexed (mm) |
| Tail length | Mean length of the tail, unsexed (mm) |
| Bill length | Mean length of the bill, unsexed (mm) |
| Tarsus length | Mean length of tarsus, unsexed (mm) |
| Weight | Mean weight in breeding season, unsexed (g) |
| Clutch size | Mean clutch size (number of eggs) |
| Broods per year | Mean number of broods per breeding season, replaced broods are not included (number of broods) |
| Egg mass | Mean weight of the egg (g) |
| Incubation period | Mean length of eggs’ incubation (days) |
| Fledging period | Mean age of young when fledging (days) |
| Age of first breeding | Mean age of the first breeding (years) |
| Life span | Maximum life span recorded in wild (years) |


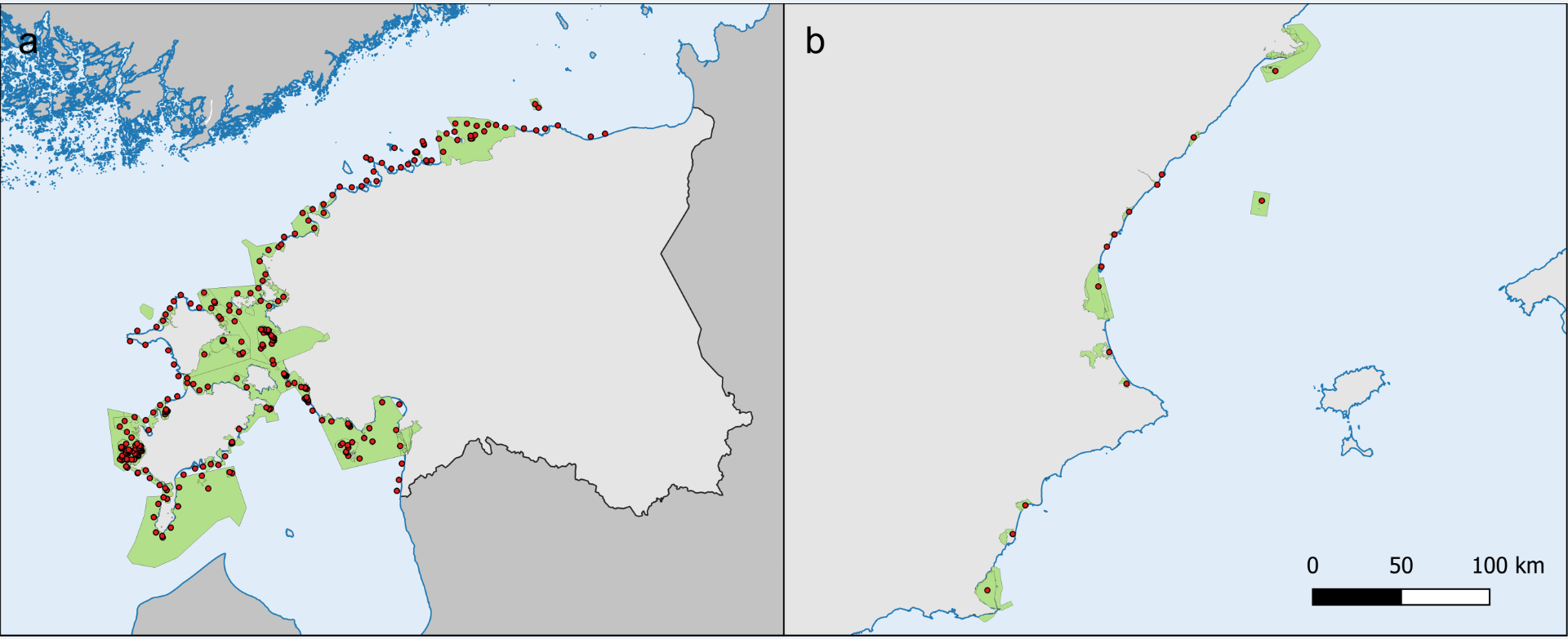


**Figure S3.** Marine and coastal protected areas in (a) the Baltic Sea (Estonia) and (b) the Western Mediterranean Sea (Spain). Only protected areas with sampling sites are shown in green.

**Table S4.** Definition of the different IUCN protected area categories.

| **Category** | **Description** |
| --- | --- |
| Ia | Strict Nature Reserve: Highly protected area allowing only minimal human activity for science, or education. |
| Ib | Wilderness area: Highly protected area managed mainly for wilderness protection. |
| II | National Park: Protected area managed for ecosystem functioning protection while allowing sustainable tourism and recreation. |
| III | Natural Monument: Protected area managed for conservation of unique natural features. |
| IV | Habitat/Species Management Area: Protected area managed for conservation of specific species or habitats through active management and restoration. |
| V | Protected Landscape/Seascape: Protected area managed for conservation of unique ecological, cultural, or scenic values while supporting sustainable human activities. |
| VI | Managed Resource Protected Area: Protected area managed for the sustainable use of particular natural resources. |

**Table S5.** Equation used to calculate the percentage of change per year.

| **Response variable** | **Transformation** | **Equation** |
| --- | --- | --- |
| Taxonomic richness | log_10_ | $Percentage of change = \left( {10}^{estimate}-1 \right) \cdot100$ |
| Taxonomic diversity | None | $Percentage of change = \frac{estimate}{mean response} \cdot100$ |
| Abundance | log_10_ | $Percentage of change = \left( {10}^{estimate}-1 \right) \cdot100$ |
| Functional richness | log_10_ | $Percentage of change = \left( {10}^{estimate}-1 \right) \cdot100$ |
| Functional evenness | None | $Percentage of change = \frac{estimate}{mean response} \cdot100$ |


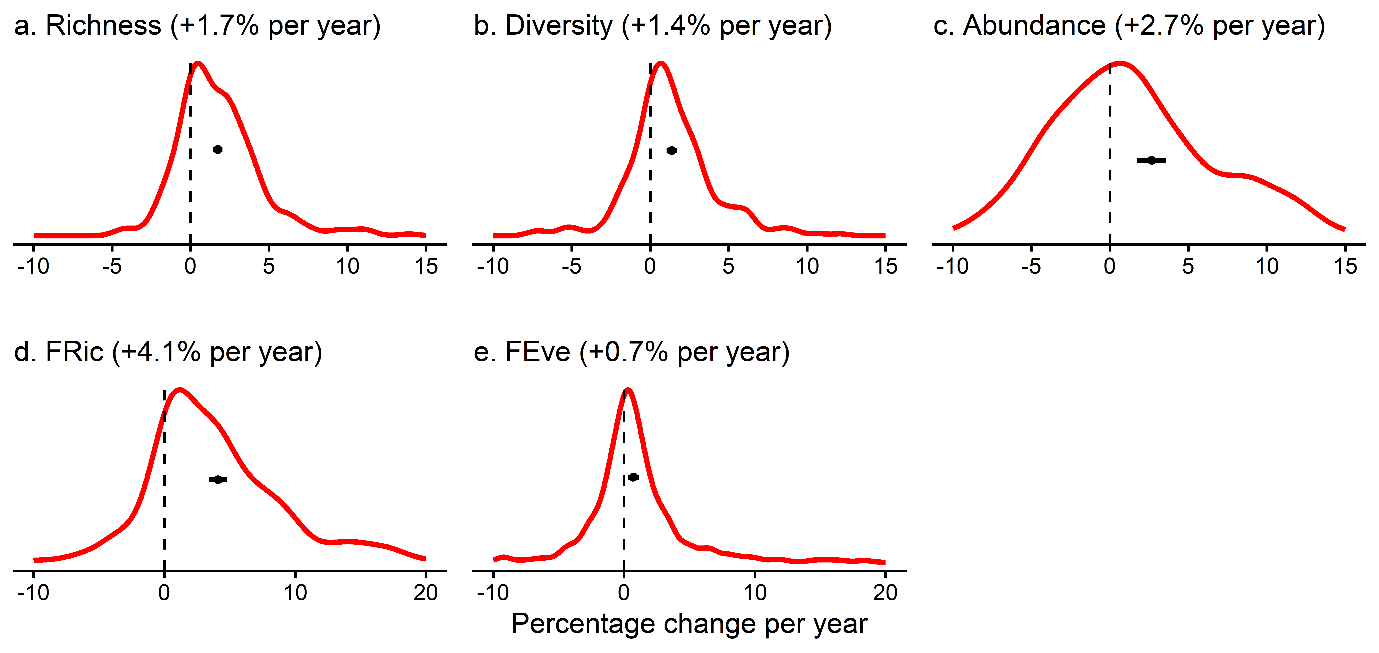


**Figure S4.** Site-level trends expressed as percentage of change per year for (a) taxonomic richness, (b) taxonomic diversity (i.e. Shannon diversity index), (c) total abundance, (d) functional richness (FRic), and (e) functional evenness (FEve) across 308 sites. The black points and text on each panel show the mean estimates (i.e. mean percentage of change per year), while the black error bars indicate the 95% confidence interval.


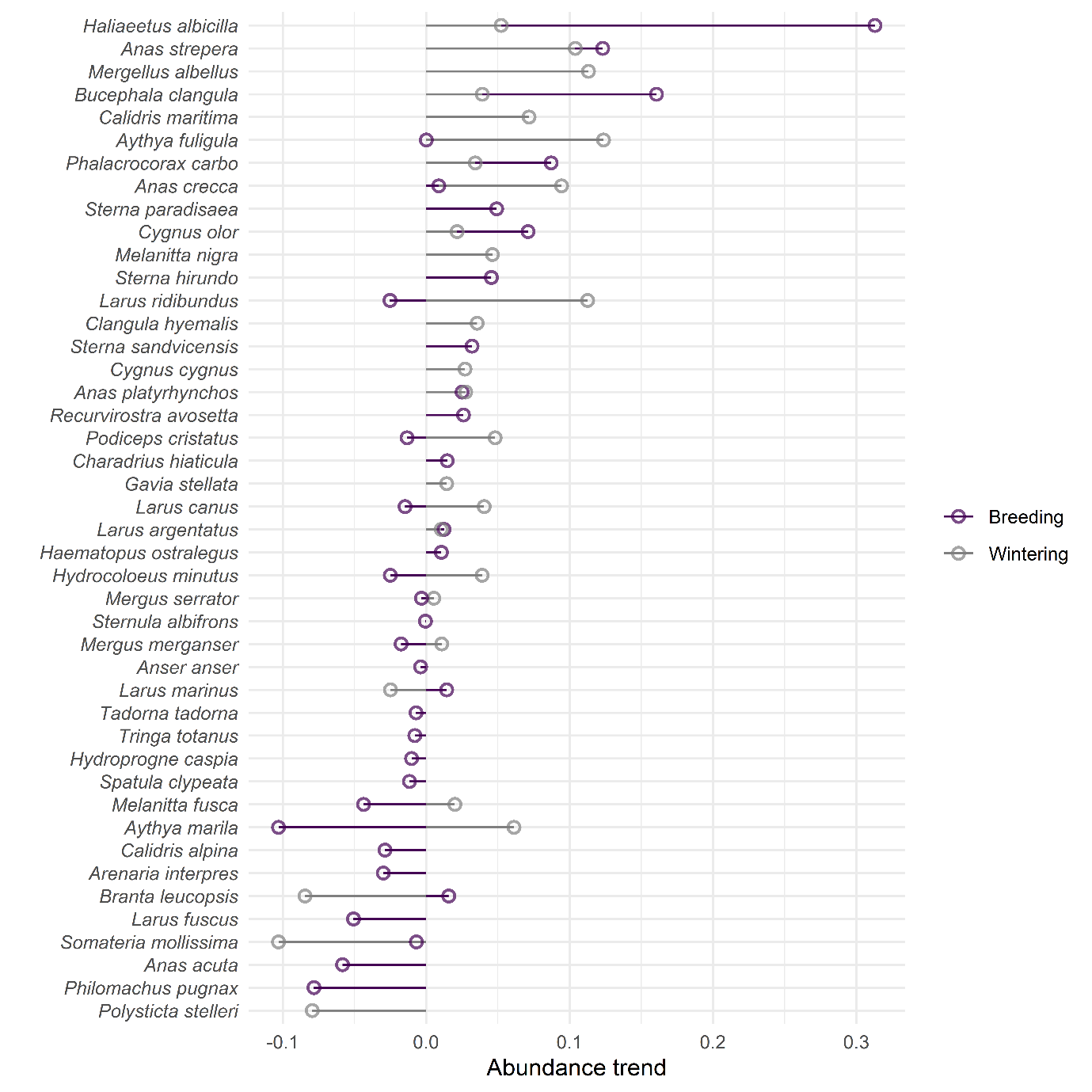


**Figure S5.** Abundance trend estimates for species sampled in the Baltic Sea.


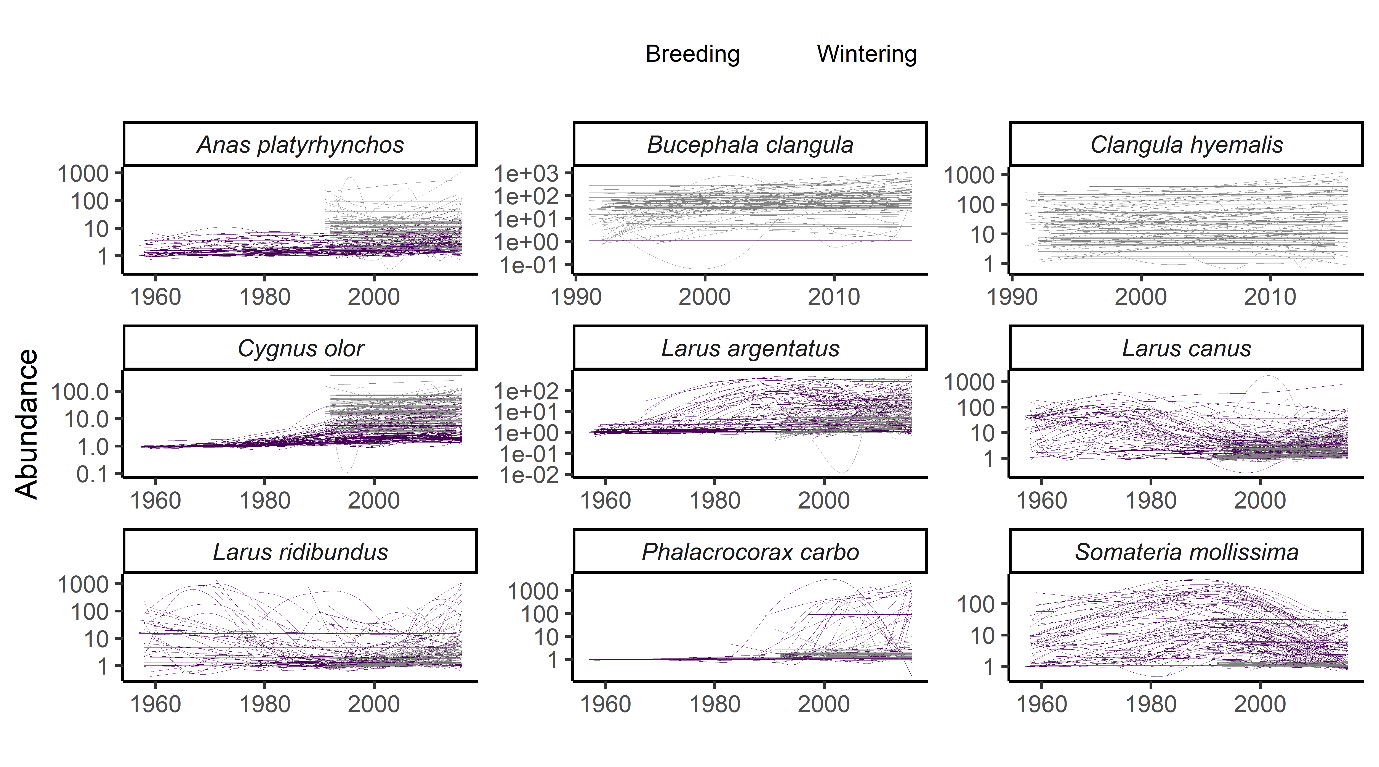


**Figure S6.** Temporal changes in abundance for the nine most common species in the Baltic Sea, based on generalized additive mixed models. Each line represents the trend at a single sampling site.


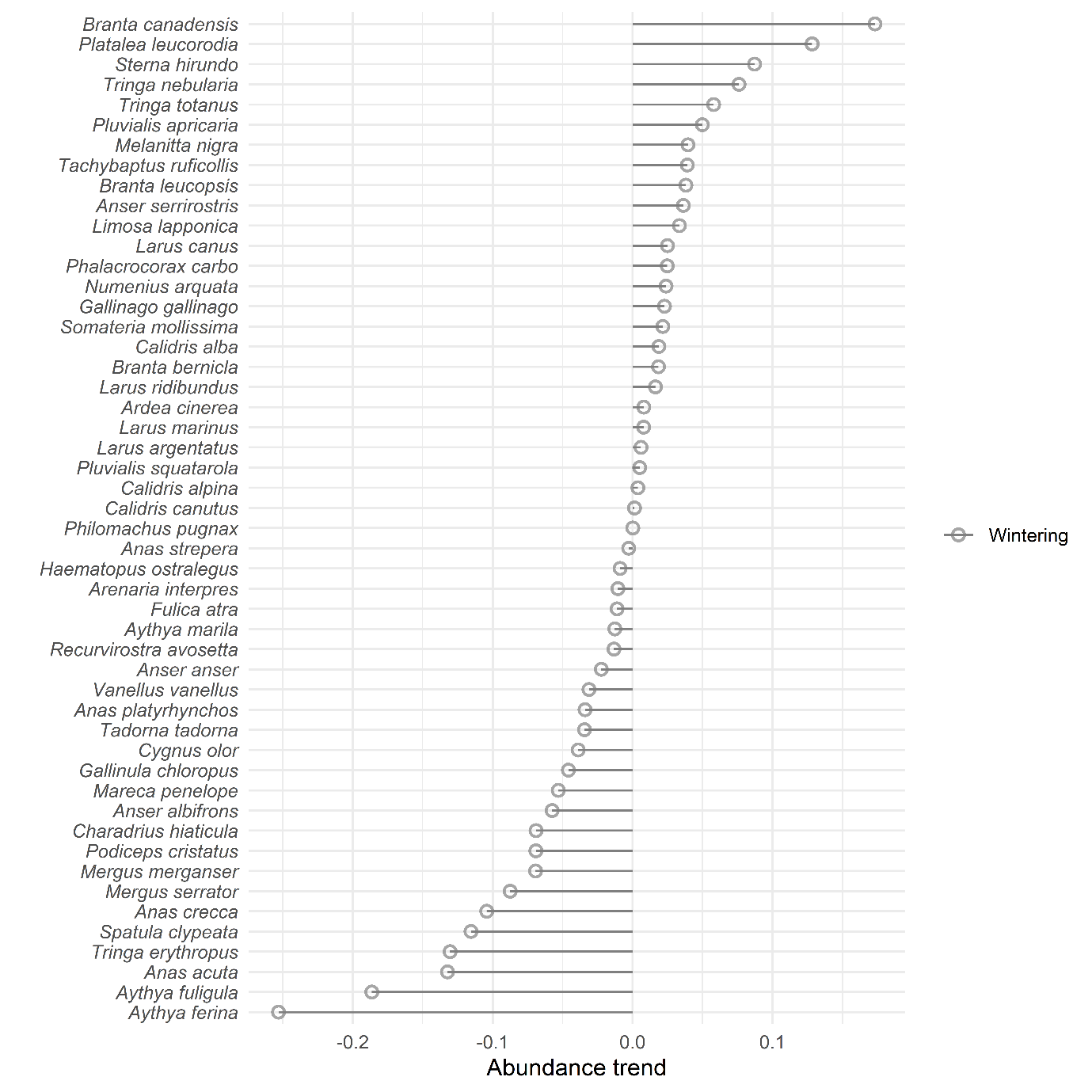


**Figure S7.** Abundance trend estimates for species sampled in the Greater North Sea.


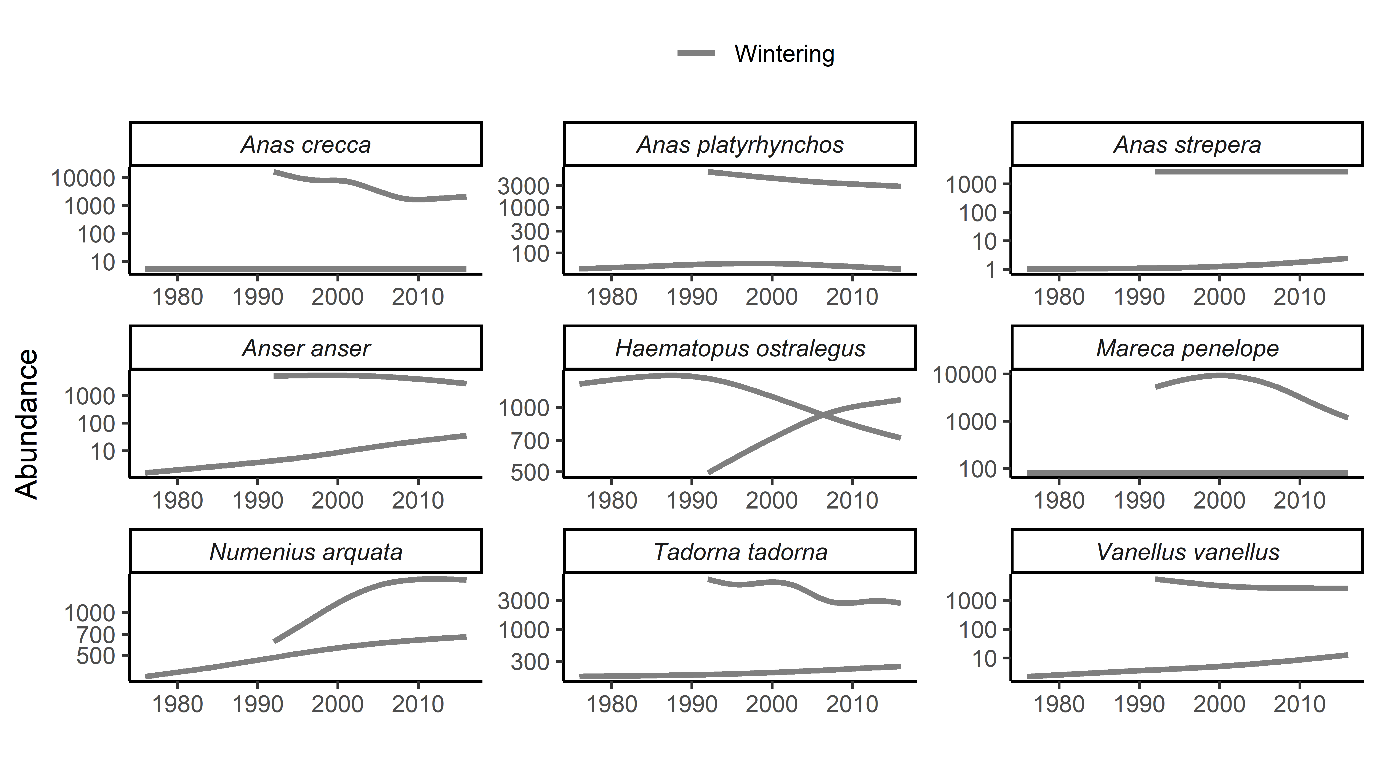


**Figure S8.** Temporal changes in abundance for the nine most common species in the Greater North Sea, based on generalized additive mixed models. Each line represents the trend at a single sampling site.


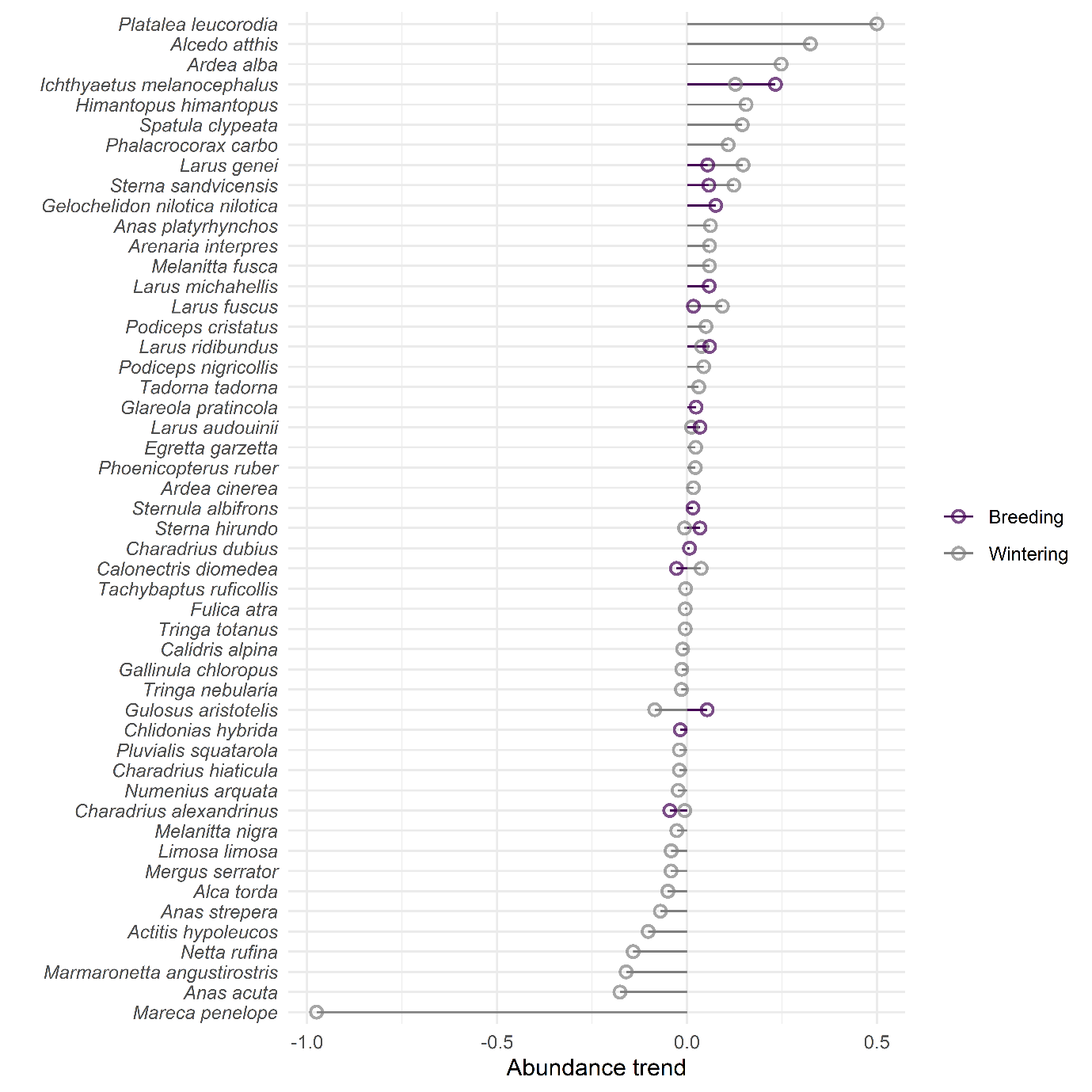


**Figure S9.** Abundance trend estimates for species sampled in the Western Mediterranean Sea.


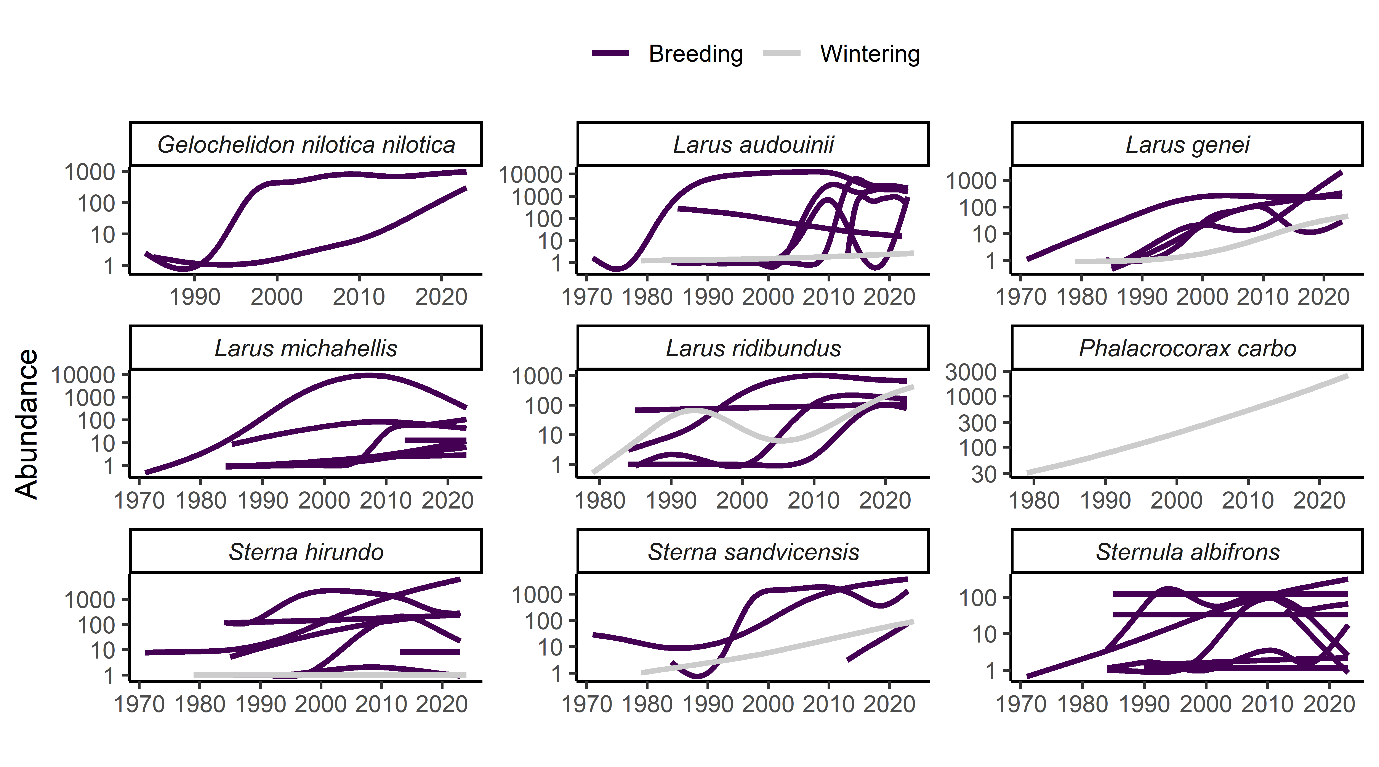


**Figure S10.** Temporal changes in abundance for the nine most common species in the Western Mediterranean Sea, based on generalized additive mixed models. Each line represents the trend at a single sampling site.

# Appendix S1: Meta-analytic multivariate model results

To test for differences in percentage of change of community metrics among regional seas, we also used a meta-analytic multivariate model, which allows to account for trend estimates uncertainty, using the function “rma.mv” of the R-package “metafor” (Viechtbauer, 2025). The results of these analyses were consistent with those presented in the main text, and showed that bird taxonomic richness increased significantly by 1.53% and 1.58% per year in the Baltic and the Western Mediterranean seas. Similarly, taxonomic diversity increased by 0.97% and 1.36% in the Baltic and the Western Mediterranean seas. In contrast, abundance only increased significantly in the Western Mediterranean Sea (4.40% per year); while functional richness only increased significantly in the Baltic Sea (3.44% per year). Changes in the Greater North Sea were not statistically significant. In addition, we did not find significant differences in biodiversity metric trends among the three regional seas (Table S6).

**Table S6.** Estimate percentage of change in biodiversity metrics for the Baltic Sea (BS), the Greater North Sea (GNS) and the Western Mediterranean Sea (WMS). Estimates significantly different from zero are highlighted in bold (* *P* ≤ 0.05; ** *P* ≤ 0.01; *** *P* ≤ 0.001). *P* values for pairwise comparisons between regions are also provided.

| **Response variable** | **BS** | **GNS** | **WMS** | **BS *vs.* GNS** | **BS *vs.* WMS** | **GNS *vs.* WMS** |
| --- | --- | --- | --- | --- | --- | --- |
| Taxonomic richness | **1.53 **** | 0.51 | **1.58 *** | 0.464 | 0.969 | 0.472 |
| Taxonomic diversity | **0.97 ***** | 0.47 | **1.36 *** | 0.996 | 0.997 | 0.998 |
| Abundance | 2.00 | -1.49 | **4.40 *** | 0.414 | 0.324 | 0.201 |
| Functional richness | **3.44 ***** | 0.09 | 1.72 | 0.174 | 0.281 | 0.526 |
| Functional evenness | 0.43 | -0.08 | 0.23 | 0.652 | 0.718 | 0.814 |

To evaluate the effect of protected areas on coastal bird communities, we also used a single linear mixed model for each metric with the following structure:

*Response variable ~ Year × Protection_Status + (1 |Sea_Name) + (1 | Site_Name)*

, where the interaction between year and the protection status is included as a fixed effect, while sea and site are included as random effects to account for regional and methodological differences among time series. The analysis revealed that all biodiversity metrics increased over time, though the magnitude of change varied across protection status. Taxonomic richness showed a greater increase in strictly protected sites compared to less protected ones, while taxonomic diversity increased more notably in strictly protected sites than in both less protected and non-protected areas. In contrast, total abundance increased more markedly in non-protected sites. No significant differences in the magnitude of change were observed for functional richness or evenness across protection status (Table S7).

**Table S7.** Results of the linear mixed models. Model estimates, standard errors, degrees of freedom (df), *t* and *P* values are shown for each explanatory variable. Marginal and conditional *R*^2^ are also shown for each model.

| **Response variable** | **Explanaroty variable** | **Estimate** | **Std. Error** | **df** | ***t*** | ***P*** |
| --- | --- | --- | --- | --- | --- | --- |
| Taxonomic richness | (Intercept) | -18.930 | 3.045 | 1852 | -6.22 | **<0.001** |
| *R*^2^_m_ = 0.031 | year | 0.010 | 0.002 | 1852 | 6.49 | **<0.001** |
| *R*^2^_c_ = 0.632 | Status Less protected | 10.520 | 4.049 | 1853 | 2.60 | **0.009** |
|  | Status Non-protected | 0.428 | 5.526 | 1873 | 0.08 | 0.938 |
|  | year:Status Less protected | -0.005 | 0.002 | 1853 | -2.58 | **0.010** |
|  | year:Status Non-protected | <0.001 | 0.003 | 1873 | -0.08 | 0.939 |
| Taxonomic diversity | (Intercept) | -40.830 | 7.150 | 1852 | -5.71 | **<0.001** |
| *R*^2^_m_ = 0.034 | year | 0.021 | 0.004 | 1851 | 5.86 | **<0.001** |
| *R*^2^_c_ = 0.524 | Status Less protected | 40.830 | 9.513 | 1853 | 4.29 | **<0.001** |
|  | Status Non-protected | 28.720 | 12.980 | 1875 | 2.21 | **0.027** |
|  | year:Status Less protected | -0.020 | 0.005 | 1852 | -4.28 | **<0.001** |
|  | year:Status Non-protected | -0.014 | 0.006 | 1875 | -2.22 | **0.027** |
| Abundance | (Intercept) | -14.960 | 8.491 | 1852 | -1.76 | 0.078 |
| *R*^2^_m_ = 0.080 | year | 0.009 | 0.004 | 1851 | 2.02 | **0.044** |
| *R*^2^_c_ = 0.662 | Status Less protected | -0.229 | 11.290 | 1852 | -0.02 | 0.984 |
|  | Status Non-protected | -122.600 | 15.430 | 1867 | -7.94 | **<0.001** |
|  | year:Status Less protected | <0.001 | 0.006 | 1852 | 0.05 | 0.962 |
|  | year:Status Non-protected | 0.061 | 0.008 | 1866 | 7.98 | **<0.001** |
| Functional richness | (Intercept) | -24.210 | 6.283 | 1815 | -3.85 | **<0.001** |
| *R*^2^_m_ = 0.013 | year | 0.012 | 0.003 | 1838 | 3.98 | **<0.001** |
| *R*^2^_c_ = 0.736 | Status Less protected | 9.600 | 8.302 | 1840 | 1.16 | 0.248 |
|  | Status Non-protected | 6.472 | 12.200 | 1885 | 0.53 | 0.596 |
|  | year:Status Less protected | -0.005 | 0.004 | 1840 | -1.14 | 0.255 |
|  | year:Status Non-protected | -0.003 | 0.006 | 1885 | -0.53 | 0.599 |

| **Response variable** | **Explanaroty variable** | **Estimate** | **Std. Error** | **df** | ***t*** | ***P*** |
| --- | --- | --- | --- | --- | --- | --- |
| Functional evenness | (Intercept) | -7.647 | 3.221 | 1841 | -2.37 | **0.018** |
| *R*^2^_m_ = 0.022 | year | 0.004 | 0.002 | 1841 | 2.51 | **0.012** |
| *R*^2^_c_ = 0.279 | Status Less protected | 6.519 | 4.263 | 1830 | 1.53 | 0.126 |
|  | Status Non-protected | 6.770 | 6.227 | 1910 | 1.09 | 0.277 |
|  | year:Status Less protected | -0.003 | 0.002 | 1830 | -1.53 | 0.125 |
|  | year:Status Non-protected | -0.003 | 0.003 | 1910 | -1.10 | 0.271 |

# References

Pilotto, F., Kühn, I., Adrian, R., Alber, R., Alignier, A., Andrews, C., Bäck, J., Barbaro, L., Beaumont, D., Beenaerts, N., Benham, S., Boukal, D. S., Bretagnolle, V., Camatti, E., Canullo, R., Cardoso, P. G., Ens, B. J., Everaert, G., Evtimova, V., … Haase, P. (2020). Meta-analysis of multidecadal biodiversity trends in Europe. *Nature Communications*, *11*(1), Article 1. https://doi.org/10.1038/s41467-020-17171-y

Viechtbauer, W. (2025). *metafor: Meta-Analysis Package for R. Version 4.8-0*.
